# Spread of multidrug resistance among *Ureaplasma* serovars, Tunisia

**DOI:** 10.1186/s13756-020-0681-5

**Published:** 2020-01-23

**Authors:** Safa Boujemaa, Béhija Mlik, Amina Ben Allaya, Helmi Mardassi, Boutheina Ben Abdelmoumen Mardassi

**Affiliations:** 1 Group of Mycoplasmas, Laboratory of Molecular Microbiology, Vaccinology, and Biotechnology Development, Institut Pasteur de Tunis, Université de Tunis El Manar, 13, Place Pasteur-B.P 74, 1002 Tunis-Belvédère, Tunisia; 2 Unit of Typing & Genetics of Mycobacteria, Laboratory of Molecular Microbiology, Vaccinology, and Biotechnology Development, Institut Pasteur de Tunis, Université de Tunis El Manar, 13, Place Pasteur-B.P 74, 1002 Tunis-Belvédère, Tunisia

**Keywords:** *Ureaplasma* spp., Biotyping, Serovars, Multidrug resistance, Tetracyclines, Fluoroquinolones, L22 mutation, Infertility

## Abstract

**Background:**

*Ureaplasma* spp. have been implicated in a variety of clinical conditions and certain serovars are likely to be disease-associated. Hence, the ascending trend of *Ureaplasma* spp. resistance to antimicrobials should deserve more attention. Here we assessed the extent of antimicrobial resistance of *Ureaplasma* serovars in Tunisia, and investigated the underlying molecular basis.

**Methods:**

This study included 101 molecularly typed *Ureaplasma* spp. clinical strains isolated over a 12-year time period (2005–2017). The antimicrobial susceptibility was tested against nine antibacterial agents using the broth microdilution method. Neighbor-joining tree was constructed to establish the phylogenetic relationships among isolates.

**Results:**

We found that all ureaplasma isolates were resistant to ciprofloxacin and erythromycin, intermediately resistant to azithromycin, and susceptible to doxycycline, moxifloxacin and josamycin. Ofloxacin and levofloxacin resistance was found in 73.27 and 17.82%, respectively, while 37.62% of isolates proved resistant to tetracycline. Consequently, we detected an elevated multidrug resistance rate among ureaplasma isolates (37.62%), particularly among serovars 2, 5, 8, and 9 (77.77% overall), as well as serovars 4, 10, 12, and 13 (52.63% overall). In most cases, drug resistance was found to be associated with known molecular mechanisms, yet we have identified two novel mutations in the L22 protein, which might be associated with macrolide-resistance.

**Conclusion:**

To our knowledge, this is the first study that reports the widespread expansion of multidrug resistance among *Ureaplasma* serovars, a finding of importance in terms of both surveillance and antimicrobial usage.

## Introduction

*Ureaplasma* spp. are members of the *Mollicutes* class of bacteria including *Ureaplasma parvum* (UPA) and *Ureaplasma urealyticum* (UUR). They are recognized as one of the smallest known self-replicating and free-living organisms. They are common inhabitants of the human genital tract, which colonization could reach 80% of healthy women in some areas of the world [1]. By contrast, ureaplasmas are less frequently isolated in the lower urogenital tract of healthy men (approximately 20–29%) [2]. The socioeconomic status, sexual activity with multiple partners, menstrual cycle, pregnancy, the use of vaginal contraceptives, and age, as well as bacterial and protozoan co-infections, favour the colonization of women genital tract by ureaplasmas [3]. Genital ureaplasmas are thought to induce a wide spectrum of pathological conditions in both men and women, including for exemple urethritis, endometritis, chronic prostatitis and bacterial vaginosis [3]. *Ureaplasma* spp. infections are undoubtedly associated with an increased risk of adverse pregnancy outcomes such as miscarriage, stillbirth, chorioamnionitis, and preterm labor [4]. They also appear to have an etiological role in a variety of severe infections in newborns such as pneumonia, bacteremia, meningitis, and chronic lung disease [5]. In addition, several investigators have presented them as causal agents of infertility [6]. In Tunisia, the role of ureaplasmas in reproductive performance impairment has been reported [7].

Currently, *Ureaplasma* spp. include 14 serovars, which are grouped into two independent species, designated UPA (serovars 1, 3, 6 and 14) and UUR (serovars 2, 4, 5 and 7–13). It has been speculated for many years that individual ureaplasma species or serovars may be associated with certain diseases more than others. Although several studies have reported that UUR is more pathogenic than UPA [8–10], conflicting results were found by others [11], so it is possible that differential pathogenicity may exist at the serovar level rather than at the species level. For example, serovar 3 was more frequently isolated from genital tract infections [7]. Similary, it has been suggested that different serovars could be responsible for different responses to antimicrobial agents [12].

Since ureaplasmas lack a cell wall structure, antibacterial agents like glycopeptides and β-lactams are completely ineffective. Likewise, ureaplasmas also have natural resistance to lincosamides (e.g. clindamycin) [13]. Hence, drugs for treatment of ureaplasma infections are confined primarily to bacteriostatic agents such as protein synthesis inhibitors (tetracyclines and macrolides), as well as, bactericidal agents that inhibit DNA replication (fluoroquinolones). As a consequence of the inappropriate use of all of the above-mentioned antibiotic classes, acquired resistance of ureaplasma isolates to these antibiotics has been documented in several studies with a rising trend, especially in developing countries [14]. Such a situation, could lead to the emergence of multidrug-resistant (MDR) strains, defined as those resistant to at least one agent in three or more antimicrobial classes [15], thus considerably restricting the treatment options**.** MDR *Mycoplasma* strains have been recently reported, particularly in *Mycoplasma genitalium* [16], while such findings are still relatively scarce in ureaplasmas [6].

The extent of antimicrobial resistance varies geographically, depending on the different antimicrobial therapy policies and the history of prior use of antimicrobial agents. To our knowledge, data regarding ureaplasma antibiotic susceptibility in Tunisia are scarce and local statistics are particularly needed to establish effective treatment. Therefore, the aim of the present study is threefold: (i) to determine the prevalence of *Ureaplasma* serovars isolated from Tunisian patients suffering from gynecological infections or infertility, (ii) to study their susceptibility patterns to various antimicrobial agents, and (iii) to investigate the underlying mechanisms of resistance.

## Material and methods

### Clinical specimens

Between years 2005 and 2017, a total of 1057 specimens from patients from Grand Tunis (in the Northeast of Tunisia) were examined by the Laboratory of Mycoplasmas of the Institut Pasteur de Tunis. The clinical specimens consisted of vaginal swabs and semen samples from patients presenting gynecological infections or infertility. The reference strains *Ureaplasma parvum* serovar 3 (UPA3) ATCC 27815, and *Ureaplasma urealyticum* serovar 8 (UUR8) ATCC 27618 were included in this study.

### *Ureaplasma* spp. isolation

All specimens were tested for the presence of *Ureaplasma* spp. by SP4-U broth and solid medium, as described by Ben Abdelmoumen Mardassi et al. [7]. As mycoplasma cultures obtained from clinical specimens can represent a mixture of several species, cloning was undertaken as detailed elsewhere [17]. Briefly, positive samples were cloned three times from single colonies recovered after plating of several dilutions.

### DNA extraction, species identification and serotyping of *Ureaplasma* spp.

Samples were prepared for PCR amplifications as described by kong et al. [18]. Briefly, cells from 2 ml of logarithmic-phase culture of each isolate were harvested by centrifugation (Heraeus Megafuge 8R, Thermo Fisher Scientific, USA) at 24,000 X *g* for 20 min. DNA was isolated by treatment with 200 μl of digestion buffer (10 mM Tris-HCl, pH 8.0; 0.45% Tween 20) and proteinase K (100 μg/ml). Lysates were incubated for 1 h at 60 °C and then for 10 min at 100 °C, and finally stored at − 20 °C. The primer sequences of the genes used in this study are displayed in Additional file 1: Table S1. Oligonucleotide primers UMS-125/UMA226 were used for amplification of the 5′-end of *mba* (Multiple Banded Antigen) gene for species identification. All amplifications were performed in a final volume of 50 μl consisted of 1 X PCR buffer (10 mM Tris-HCl, pH 8.3; 50 mM KCl) (Invitrogen, USA), 2.5 mM MgCl_2_ (Invitrogen, USA), 0.4 μM of each primer (Sigma Genosys, Germany), 0.2 mM dNTPs (Sigma-Aldrich, Germany), 1.25 units of *Taq* DNA polymerase (Invitrogen, USA) and 10 μl of treated sample. An Applied Biosystems Thermal Cycler 2720 (Life Technologies, USA) was set up with a first cycle of denaturation for 5 min at 94 °C, followed by 35 cycles of denaturation at 94 °C for 1 min, annealing at 60 °C for 1 min, elongation at 72 °C for 1 min, and a final extension step of 7 min at 72 °C. The PCR products were electrophoresed on 2% agarose gel and stained with ethidium bromide (Sigma-Aldrich, Germany) to visualize the DNA bands, whose sizes were determined using a 100-bp DNA ladder (Invitrogen, USA). Based on the amplicon size, *Ureaplasma* strains were assigned to UPA (403 or 404 bp) or UUR (448 bp) [19]. The PCR products of the 5′-end of *mba* gene of all strains were sequenced for serotyping characterization of *Ureaplasma* serovars. Briefly, PCR products were purified with both Exonuclease I (Biolabs, England) and Shrimp Alkaline Phosphatase (Biolabs, England) as per manufacturer’s instructions. Sequencing reactions were performed using a BigDye Terminator v3.1 cycle sequencing kit (Applied Biosystems, USA), according to the manufacturer’s recommendations, and were run in an ABI Prism 3130 genetic analyzer (Applied Biosystems, USA). Both strands from each PCR amplicon were sequenced twice. The nucleotide sequences obtained were aligned with reference sequences of 14 ATCC *Ureaplasma* serovars, that were retrieved from GenBank as follows: ABES01000000 (UPA1), ABFL02000000 (UUR2), CP000942.1 (UPA3), AAYO02000000 (UUR4), AAZR01000000 (UUR5), AAZQ01000000 (UPA6), AAYP01000000 (UUR7), AAYN02000000 (UUR8), AAYQ02000000 (UUR9), CP001184 (UUR10), AAZS01000000 (UUR11), AAZT01000000 (UUR12), ABEV01000000 (UUR13) and ABER01000000 (UPA14). The alignment was conducted by using BioEdit Sequence Alignment Editor version 7.2.5 [20].

### Phylogenetic analysis

The 5′-end of *mba* gene sequences of all clinical isolates (403/404 bp for UPA and 448 bp for UUR) were phylogenetically analyzed by using MEGA version 6.06 [21]. The evolutionary distances were estimated by using Kimura’s two-parameter method, and a circular phylogenetic tree was constructed using the neighbor-joining method. Bootstrap analyses were performed by 1000 resamplings of the data sets.

### Antimicrobial agents

The susceptibility tests included nine antimicrobial agents: tetracyclines (tetracycline ‘TET’, doxycycline ‘DOX’); fluoroquinolones (ofloxacin ‘OFX’, ciprofloxacin ‘CIP’, levofloxacin ‘LVX’, moxifloxacin ‘MXF’); and macrolides (erythromycin ‘ERY’, azithromycin ‘AZM’ and josamycin ‘JOS’) (Sigma-Aldrich, Germany). Each antimicrobial agent was prepared according to the manufacturer’s instructions at a stock concentration of 1280 mg/L and stored at −20°C before use.

### Antimicrobial susceptibility testing

Antimicrobial susceptibility was determined using a broth microdilution method following the Clinical Laboratory Standards Institute (CLSI) guidelines [22]. The reference type strains UPA3 ATCC 27815 and UUR 8 ATCC 27618 were included in each test to ensure quality control. The specific breakpoints (mg/L) indicating susceptibility (S) or resistance (R) are: tetracycline S ≤ 1, R ≥ 2; doxycycline S ≤ 4, R ≥ 8; ciprofloxacin S ≤ 1, R ≥ 2; ofloxacin S ≤ 1, R ≥ 4; levofloxacin S ≤ 2, R ≥ 4; moxifloxacin S ≤ 2, R ≥ 4; erythromycin S ≤ 8, R ≥ 16; azithromycin S ≤ 0.125, R ≥ 4; and josamycin S ≤ 2, R ≥ 8. Since interpretative criteria for DOX, OFX, CIP, JOS, and AZI are not available from CLSI, the minimum inhibitory concentration (MIC) values were interpreted according to the Mycoplasma IST-2 kit (bioMerieux, Marcy-l’Etoile, France) criteria.

### Resistance genes amplification and sequencing

*tet* (M) and *Int-Tn* genes were amplified from both susceptible- and resistant-strains as previously detailed [16]. For fluoroquinolone-resistant strains, the quinolone resistance determining regions (QRDRs) of *gyrA*, *gyrB*, *parC*, and *parE* genes were studied by PCR amplification and sequencing [23]. Simultaneously, macrolide-resistant strains underwent PCR amplification and sequencing of both 23S rRNA alleles, as well as the genes encoding ribosomal proteins L4 and L22 [24]. Amplicons were then purified and sequenced as detailed above. Sequences were analyzed using BioEdit and translated into amino acids (http://www.expasy.org/). The amino acid sequences of GyrA, GyrB, ParC, ParE, L4, and L22 proteins, as well as the nucleotide sequences of the 23S rRNA genes were compared with the respective sequences of the 14 ATCC *Ureaplasma* serovars, in order to distinct species- or serovar-specific polymorphisms from those associated with resistance.

### Statistical analyses

Statistical analyses were conducted using GraphPad Prism for Windows v. 7.03. The statistical tests applied in this study were the Chi-Square test and t-test. A *P* value < 0.05 was considered as statistically significant.

## Results

### Prevalence of *Ureaplasma* spp. in the urogenital tract

*Ureaplasma* spp. were detected in high titers (≥ 10^4^ CFU/ml) in 101 (9.55%) out of 1057 cultured samples. Out of these, 85 isolates (84.2%) were recovered from infertile patients and 16 strains (15.8%) from patients suffering from gynecological infections (Additional file 1: Table S2).

All specimens found to be positive for *Ureaplasma* spp. were biotyped by *mba*-based PCR. Of the 101 *Ureaplasma* isolates, UPA and UUR were found in 73 (72.28%) and 28 (27.72%) patients, respectively. None of the patients was coinfected with both species. UPA- and UUR- positive isolates were further subtyped into different serovars by nucleotide sequencing. Among UPA strains, serovar 3 was the most frequent (35 out of 73, 47.94%), followed by serovars 1 and 6 (19 out of 73 for each one, 26.03%). Subtyping of UUR from the 28 positive cases showed serovars 4, 10, 12, and 13 in 19 cases (67.86%) and serovars 2, 5, 8 and 9 in 9 cases (32.14%). No UPA serovar 14 or UUR serovars 7 and 11 were detected (Additional file 2: File S1, File S2, File S3, File S4 and File S5).

### Correlation between *Ureaplasma* serovars and gender

The positive rate of ureaplasma infections was higher in female, who were in the reproductive age (25–40 years old) (76 cases), than male (25 cases). By contrast, the distribution of different *Ureaplasma* serovars showed no statistically significant difference between the two genders (*P* = 0.7835).

### Correlation between *Ureaplasma* serovars and clinical manifestations

The positive rate of *Ureaplasma* spp. was higher in infertile patients (85 of 101, 84.2%) then patients suffering from gynecological infections (16 of 101, 15.8%). The distribution of *Ureaplasma* serovars among patients with different clinical status showed a statistically significant difference (*P* = 0.0102). In patients with genital tract infections, serovar 1 was predominant in 8 cases (50%) while in infertile patients, serovar 3 was predominant in 29 cases (34.12%). Interestingly, we observed that UUR 2, 5, 8, 9, and UUR 4, 10, 12, 13 clinical isolates were exclusively recovered from infertile patients.

### Antimicrobial susceptibility patterns over the past 12 years

A summary of the MIC results of the 101 *Ureaplasma* spp. clinical isolates is shown in Additional file 1: Table S3.

**Fig. 1:**
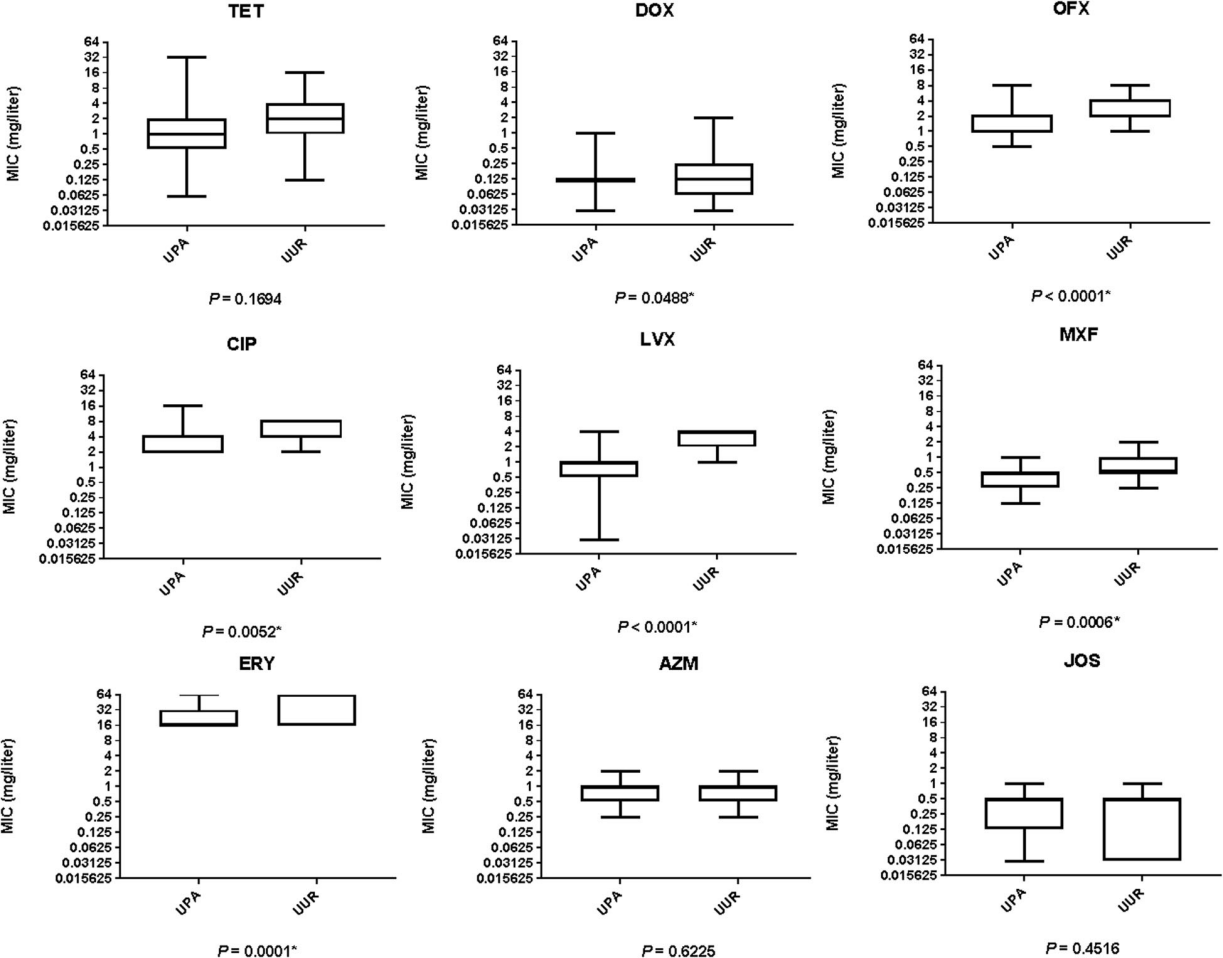
Boxplots comparing MICs for each antibiotic tested between *U*. *parvum* (UPA) and *U. urealyticum* (UUR) isolates. Interquartile and median MIC values are represented by boxes and centerline, respectively. Lower and upper whiskers represent minimum and maximum values, respectively. * Represents there is significant difference between the species

When comparing MIC data between species, UUR isolates had significantly higher MIC values compared to UPA isolates against DOX, OFX, CIP, LVX, MXF, and ERY (Fig. 1). By contrast, no significant difference between the two species was observed in the MICs of TET, AZM, and JOS (*P* > 0.05).

**Fig. 2:**
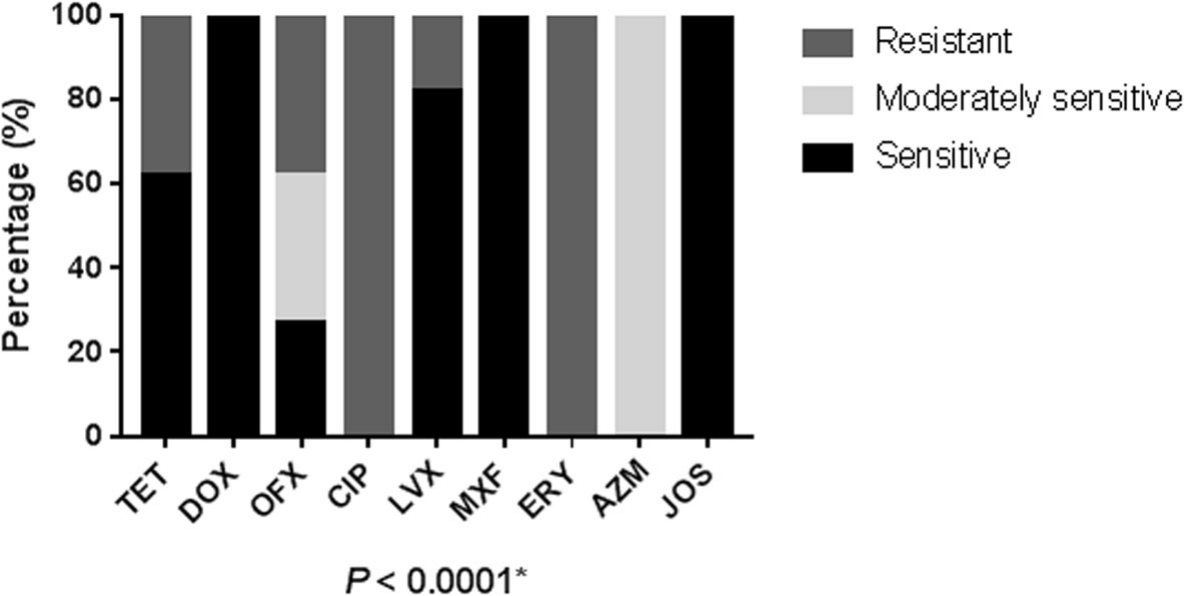
Antimicrobial susceptibility of *Ureaplasma* spp. strains to the antibacterial agents. * Represents there is significant difference

Regardless to serovars, statistical analysis showed a significant difference in the susceptibility rates of *Ureaplasma* spp. strains to different antibacterial agents (*P* < 0.0001). Indeed, all *Ureaplasma* spp. isolates were: sensitive to DOX, MXF, and JOS (MICs ≤1 mg/L); moderately sensitive to AZM (0.25 ≤ MICs ≥2 mg/L); and resistant to ERY (MICs ≥16 mg/L) and CIP (MICs ≥4 mg/L). Regarding tetracycline susceptibility, 63 (62.38%) strains were found sensitive. The examination of susceptibility to fluoroquinolones indicated that 27 (26.73%) strains were sensitive and 35 (34.65%) were moderately sensitive to OFX. Yet, *Ureaplasma* spp. strains showed a high sensitivity rate to LVX (83/101, 82.18%) (Fig. 2). In addition, we noticed that the susceptibility rate to TET, OFX and LVX remained almost constant throughout the studied time period (*P* = 0.1917).

### Sex-, clinical status-, and serovar-specific trends in antibiotic resistance patterns of *Ureaplasma* spp.

Compared with strains isolated from male patients, strains isolated from female patients displayed slightly higher resistance rates to LVX and OFX, as well as slightly lower resistance rate to TET.

Strains isolated from infertile patients displayed a significant higher resistance rate to TET and a significant higher sensitivity rate to OFX compared to those isolated from patients presenting with genital infections (Additional file 1: Table S4). However, the observed difference in resistance rates to LVX between the two clinical manifestations was not statistically significant.

**Fig. 3:**
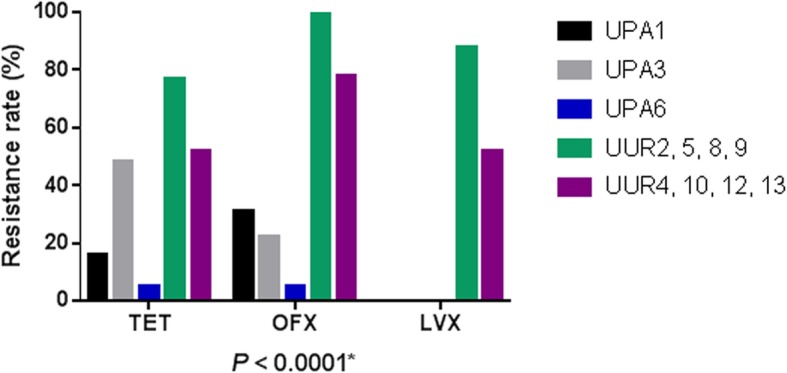
Antibiotic resistance rates of *Ureaplasma* serovars to TET, OFX, and LVX. * Represents there is significant difference among the serovars

Interestingly, *Ureaplasma* serovars showed a significant difference in their resistance patterns to TET, OFX, and LVX (*P* < 0.0001) (Fig. 3). Indeed, UUR strains showed more resistance to TET, OFX, and LVX (60.71, 85.71, and 64.29%, respectively) than did UPA strains (28.77, 20.55, and 0%, respectively) (*P* < 0.0001). UUR2, 5, 8, 9 strains displayed the highest resistance rates to TET (77.77%), OFX (100%) and LVX (88.88%). However, UPA6 strains were the most sensitive with a resistance rate of 5.26, 5.26, and 0% to TET, OFX, and LVX, respectively.

### Clustering of multidrug-resistant strains

**Fig. 4:**
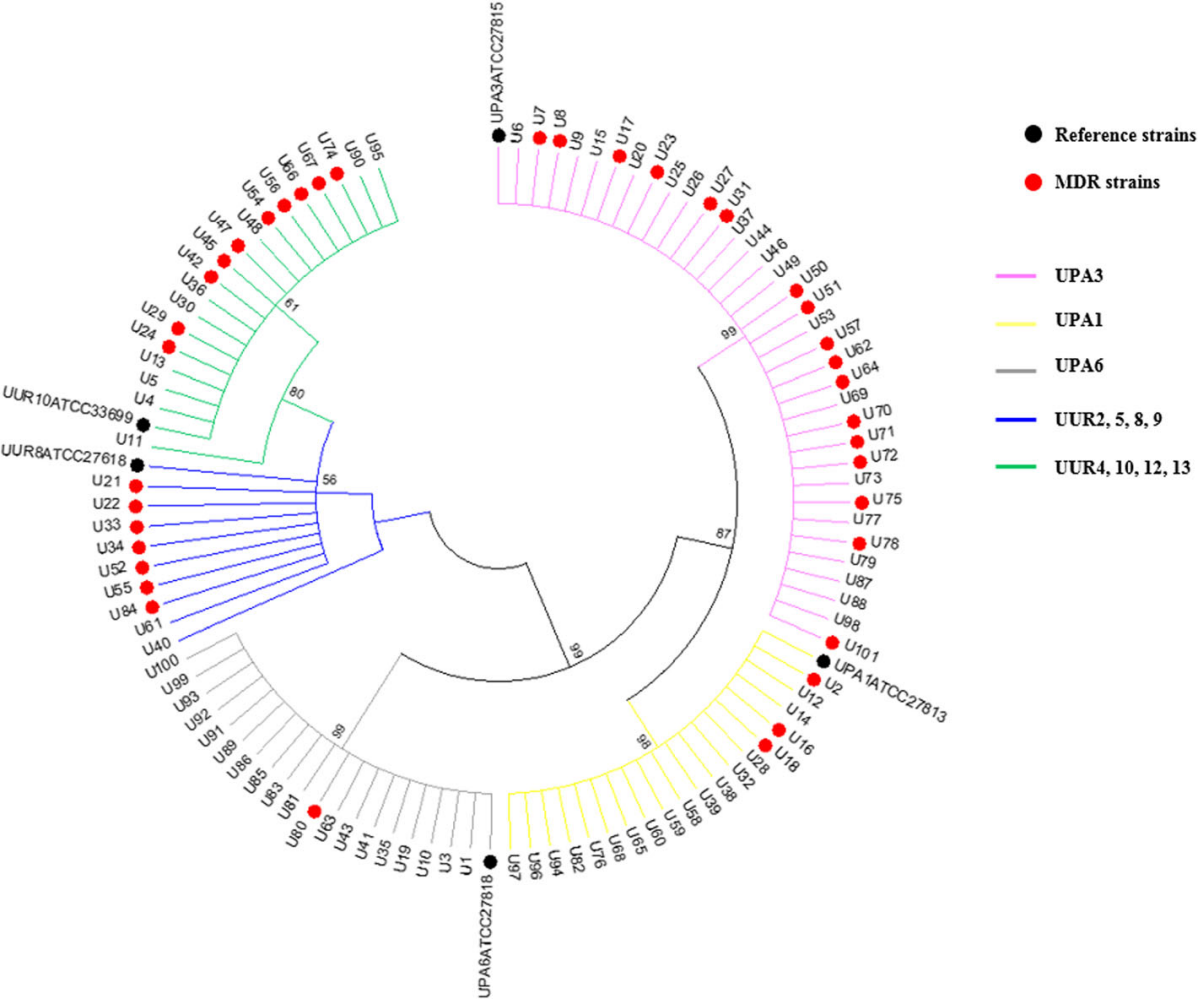
Neighbor-joining tree of 101 *Ureaplasma* spp. isolates based on the 5′-end of *mba* gene sequences. The branches are colored according to the serovar. Black circles correspond to reference strains and MDR isolates are represented by red circles

The NJ tree based on the 5′-end sequence of *mba* gene clearly distinguished five clusters, which corresponded to UPA3, UPA6, UPA1, UUR2, 5, 8, 9 and UUR4, 10, 12, 13 (Fig. 4). Overall, 37.62% of the strains were MDR, with 60.71% (17/28) of UUR strains and 28.76% (21/73) of UPA being MDR. Regarding serovars, the majority of UUR2, 5, 8, 9 (77.77%, 7/9) were MDR followed by UUR4, 10, 12, 13 (52.63%, 10/19), and UPA3 (48.57%, 17/35). However, 15.79% of UPA1 (3/19) and only one isolate of UPA6 were MDR.

### Molecular analysis of genetic mechanisms of resistance

#### Tetracyclines

Screening of the *tet* (M) determinant, which is associated with tetracycline resistance, as well as the *Int-Tn* gene, revealed that all tetracycline-resistant *Ureaplasma* spp. clinical isolates, simultaneously, harboured these two genes (Additional file 1: Figure S5). All the PCR products of *tet* (M) and *Int-Tn* genes migrated at the expected sizes of 397 bp and 579 bp, respectively. Neither *tet* (M) nor *Int-Tn* sequences could be amplified from tetracycline-sensitive clinical isolates.

#### Fluoroquinolones

The nucleotide and deduced amino acid sequences of the QRDRs of *gyrA*, *gyrB*, *parC* and *parE* for a selection of clinical *Ureaplasma* spp. strains were compared with the respective 14 ATCC *Ureaplasma* serovars, and thus species- or serovar-specific polymorphisms could be avoided.

Sequence comparison revealed no mutations in any of the sequenced isolates within either *gyrA* or *gyrB* genes; however, mutations were observed in the *parC* and *parE* genes. A single base pair change (C248T) in the *parC* gene was identified in all levofloxacin-resistant strains, resulting in the substitution of serine by leucine at position 83 (S83L) (Additional file 2: File S6). A point mutation at position 1336 (C1336T) in the *parE* gene was identified in three levofloxacin- resistant strains, resulting in the proline-to-serine substitution at position 446 (P446S) (Additional file 2: File S7). For the above clinical strains, which harboured the double mutation S83L in ParC and P446S in ParE, the MICs of the four fluoroquinolones were higher (OFX: 8 mg/L, CIP: 8 mg/L, LVX: 4 mg/L, MXF: 2 mg/L) compared to strains bearing the single mutation S83L in ParC. However, no mutation was detected in four ciprofloxacin-resistant strains (MIC: 4 mg/L). Detailed results of the genes responsible for antimicrobial resistance are summarized in Table 1.

**Table 1:** Molecular characterization of antibiotic resistance mechanisms of 26 *Ureaplasma* spp. isolates

Strain	Serovar	MICs (mg/liter)^a^							QRDRs analysis^b^		Macrolide resistance trait^b^
		OFX	CIP	LVX	MXF	AZM	ERY	JOS	ParC	ParE	L22
ATCC 27815	UPA3	0.25 (S)	0.5 (S)	0.125 (S)	0.06 (S)	0.06 (S)	2 (S)	0.03 (S)	WT	WT	WT
ATCC 27618	UUR8	0.5 (S)	0.5 (S)	0.25 (S)	0.06 (S)	0.06 (S)	2 (S)	0.03 (S)	WT	WT	WT
U1	UPA6	2 (MS)	8 (R)	1 (S)	0.5 (S)	1 (MS)	64 (R)	0.5 (S)	S83L	WT	A121S, T141I
U2	UPA1	2 (MS)	4 (R)	1 (S)	0.5 (S)	0.5 (MS)	16 (R)	0.5 (S)	WT	WT	A121S, T141I
U4	UUR4, 10, 12, 13	2 (MS)	4 (R)	2 (S)	0.5 (S)	1 (MS)	64 (R)	0.03 (S)	WT	WT	A121S, T141I
U6	UPA3	4 (R)	4 (R)	0.25 (S)	0.5 (S)	0.25 (MS)	16 (R)	0.125 (S)	WT	WT	A121S, T141I
U10	UPA6	4 (R)	8 (R)	1 (S)	0.5 (S)	0.5 (MS)	16 (R)	0.5 (S)	S83L	WT	A121S, T141I
U11	UUR4, 10, 12, 13	4 (R)	4 (R)	4 (R)	1 (S)	1 (MS)	32 (R)	0.03 (S)	S83L	WT	A121S, T141I
U13	UUR4, 10, 12, 13	4 (R)	8 (R)	4 (R)	0.5 (S)	0.25 (MS)	64 (R)	0.03 (S)	S83L	WT	A121S, T141I
U14	UPA1	8 (R)	16 (R)	1 (S)	0.5 (S)	2 (MS)	64 (R)	0.5 (S)	S83L	WT	A121S, T141I
U15	UPA3	8 (R)	8 (R)	0.5 (S)	0.5 (S)	1 (MS)	16 (R)	0.5 (S)	S83L	WT	A121S, T141I
U16	UPA1	1 (S)	4 (R)	0.5 (S)	0.25 (S)	0.5 (MS)	16 (R)	0.125 (S)	WT	WT	A121S, T141I
U21	UUR2, 5, 8, 9	8 (R)	8 (R)	4 (R)	2 (S)	1 (MS)	64 (R)	0.25 (S)	S83L	P446S	A121S, T141I
U22	UUR2, 5, 8, 9	4 (R)	4 (R)	4 (R)	1 (S)	2 (MS)	16 (R)	0.5 (S)	S83L	WT	A121S, T141I
U24	UUR4, 10, 12, 13	4 (R)	4 (R)	4 (R)	0.5 (S)	1 (MS)	64 (R)	0.03 (S)	S83L	WT	A121S, T141I
U30	UUR4, 10, 12, 13	4 (R)	8 (R)	4 (R)	0.5 (S)	1 (MS)	32 (R)	0.03 (S)	S83L	WT	A121S, T141I
U33	UUR2, 5, 8, 9	4 (R)	4 (R)	4 (R)	0.5 (S)	1 (MS)	64 (R)	1 (S)	S83L	WT	A121S, T141I
U34	UUR2, 5, 8, 9	4 (R)	8 (R)	4 (R)	0.5 (S)	0.5 (MS)	16 (R)	1 (S)	S83L	WT	A121S, T141I
U36	UUR4, 10, 12, 13	4 (R)	8 (R)	4 (R)	0.5 (S)	0.25 (MS)	64 (R)	0.03 (S)	S83L	WT	A121S, T141I
U40	UUR2, 5, 8, 9	4 (R)	4 (R)	4 (R)	0.5 (S)	0.5 (MS)	64 (R)	0.5 (S)	S83L	WT	A121S, T141I
U47	UUR4, 10, 12, 13	4 (R)	4 (R)	4 (R)	1 (S)	0.5 (MS)	16 (R)	0.5 (S)	S83L	WT	A121S, T141I
U52	UUR2, 5, 8, 9	4 (R)	8 (R)	4 (R)	0.5 (S)	1 (MS)	64 (R)	0.5 (S)	S83L	WT	A121S, T141I
U54	UUR4, 10, 12, 13	4 (R)	8 (R)	4 (R)	1 (S)	2 (MS)	64 (R)	0.5 (S)	S83L	WT	A121S, T141I
U55	UUR2, 5, 8, 9	8 (R)	8 (R)	4 (R)	2 (S)	2 (MS)	64 (R)	0.5 (S)	S83L	P446S	A121S, T141I
U56	UUR4, 10, 12, 13	4 (R)	4 (R)	4 (R)	1 (S)	2 (MS)	64 (R)	0.25 (S)	S83L	WT	A121S, T141I
U66	UUR4, 10, 12, 13	4 (R)	4 (R)	4 (R)	1 (S)	0.5 (MS)	16 (R)	0.5 (S)	S83L	WT	A121S, T141I
U67	UUR4, 10, 12, 13	4 (R)	4 (R)	4 (R)	1 (S)	2 (MS)	64 (R)	0.25 (S)	S83L	WT	A121S, T141I
U84	UUR2, 5, 8, 9	8 (R)	8 (R)	4 (R)	2 (S)	1 (MS)	64 (R)	0.5 (S)	S83L	P446S	A121S, T141I

^a^*S* Sensitive, *MS* Moderately sensitive, *R* Resistant, ^b^*WT* Wild-type

#### Macrolides

The 23S rRNA genes and associated L4 and L22 proteins were sequenced from 26 ureaplasma isolates resistant to ERY (MICs ≥16 mg/L) (Table 1). No mutations were found in either the 23S rRNA operons or the L4 protein of any of the isolates, yet two new point mutations (G361 T and C422T) were detected in the L22 gene of all isolates studied that translated into amino acid substitutions A121S and T141I, respectively (Additional file 2: File S8).

## Discussion

Compared to the rate of multidrug resistance recently reported by Zhu et al. [6], our study is reporting a higher rate among clinical strains of ureaplasmas. Indeed, the prevalence of MDR *Ureaplasma* spp. strains tested herein was particularly high (37.62%), which is likely to be the result of the widespread uncontrolled and increased misuse of antibacterial agents in Tunisia, a phenomenon that has been previously stressed [25, 26].

In line with previous reports, this study showed higher MIC trends exhibited by UUR compared to UPA [27, 28], suggesting a possible inherently increased tolerance of UUR isolates to drugs relative to that of UPA isolates, particularly serovars 2, 5, 8, 9. However, differences in susceptibilities, with sometimes conflicting results, have been reported between the two ureaplasma species and within the 14 distinct serovars [12]. Unexpectedly, we found an elevated multi-drug resistance rate in UPA3. Given that all the isolates were uniformely resistant to CIP and ERY, this result could be explained by the high resistance rate of UPA3 to TET.

Tetracyclines represent the first-line treatment of infections caused by *Ureaplasma* spp., to which resistance is continuously increasing. Herein, *Ureaplasma* spp. strains showed a reduced susceptibility to TET (62.38%), but an extremely high sensitivity to DOX, as reported previously [29]. This rate is fairly consistent with that reported by Díaz et al. in Cuba [30], but distinctly different to a study from France, which reported a higher (>80%) sensitivity rates to TET [31].

High percentages of resistance to fluoroquinolones was demonstrated, most likely as a consequence of their more frequent use for plenty of infections. Hitherto, it was widely reported that CIP and OFX were mainly inactive [14]. However, the newer fluoroquinolones, LVX and MXF presented a greater effectiveness against *Ureaplasma* spp., as described elsewhere [32]. By contrast, Song et al. have reported in China a higher resistance rate to LVX (76.9%) and MXF (23.1%), compared to our findings (17.82 and 0%, respectively) [33]. In our study, all isolates were resistant to ERY and moderately sensitive to AZM, which was surprising as *Ureaplasma* spp. is known to be susceptible to macrolides [34]. Yet, a study conducted in South Africa has reported a high resistance rate to ERY, reaching 80% [13]. Similarly, AZM-resistant strains of *Ureaplasma* spp. are now being reported with increasing frequency [12]. No resistance was seen against the new macrolide, JOS for *Ureaplasma* spp*.*, which is in agreement with earlier studies [14, 29]. Remarkably, the antibacterial resistance profiles of ureaplasma isolates from different geographical locations vary significantly, which might be the result of variations in antimicrobial exposure in different patient populations. In some developing countries, the overuse and/or inappropriate use of antibiotics are relatively common so that resistance patterns may exceed what is seen in the industrialized world [35]. This emphasizes the need for local surveillance of antimicrobial resistance in these countries in order to guide empirical treatment.

Currently, the only known mechanism of tetracycline resistance for *Mollicutes* is the presence of the *tet* (M) transferable genetic element. In this study, we demonstrated that our clinical tetracycline-resistant isolates harbored DNA sequences related to the *tet* (M) determinant and the *Int-Tn* gene. Previous study conducted in our laboratory has showed that the nucleotide sequence analysis of the *tet* (M) amplicon revealed a unique sequence shared by all tetracycline-resistant clinical isolates of both UPA and *Mycoplasma hominis*, suggesting that it may have benefited from horizontal gene transfer, making it highly competent to spread [7].

It seems that fluoroquinolone abuse leads to resistance, mainly through target enzyme mutations. Here, we identified the most common amino acid substitution (S83L) in the ParC protein, which was found in 22 of 26 fluoroquinolone-resistant isolates. This is in agreement with many prior reports indicating that S83L is the most frequently mutation described thus far in *Ureaplasma* fluoroquinolone-resistant isolates [27]. This mutation is located in the proposed active site of the ParC protein, and is homologous to the mutation identified in other fluoroquinolone-resistant bacteria (S80L), such as *Acinetobacter baumannii* and *Pseudomonas aeruginosa* [36]. However, we noted that 4 of 26 fluoroquinolone-resistant isolates studied had no amino acid substitutions in the QRDRs. This finding is not without precedent and could be linked to mutations outside the sequenced regions or alternative mechanisms, not yet elucidated, such as altered membrane permeability [37]. An atypical substitution in the QRDR region of the ParE protein, P446S, was detected in only three resistant isolates, which also harbored the S83L substitution. Although P446S was not previously associated with fluoroquinolone resistance in other bacteria, we suggest that it might lead to the increased resistance to fluoroquinolone observed for these three isolates. In agreement to this finding, Chang et al. have recently detected P446S substitution in one ofloxacin-resistant isolate [38]. Interestingly, the same substitution was found also in a highly moxifloxacin-resistant *Mycoplasma genitalium* strain, which harboured also S83L substitution in ParC protein [39].

With regard to macrolide resistance, we detected two amino acid substitutions in the L22 protein, A121S and T141I, for all the macrolide-resistant strains studied. Both substitutions were previously described in a highly macrolide-resistant strain, but it has been concluded that they may not contribute to macrolide resistance because of the presence of a five-amino acid insertion (69TGKAR70) in the extended loop of the L4 protein, which has been also described in *Streptococcus pneumoniae* [40]. Yet, additional mutations outside of the investigated regions, DNA methylation, or increased drug efflux may also be responsible for the relative increase in tolerance to erythromycin for these strains. In light of the epidemiological characteristics of ureaplasma strains, the present results may aid Tunisian clinicians to implement rational drug usage that would prevent and control the spread of antibiotic resistance.

## Conclusion

In sum, this study provides a detailed picture on the genetic diversity, drug susceptibility, and resistance mechanisms in *Ureaplasma* spp. clinical isolates, an investigation that has not previously been drawn up, in Tunisia. Our study shows that *Ureaplasma* spp. isolation is frequent among infertile patients, and highlights for the first time the emergence of a high rate of MDR strains. Based on these findings, DOX, MXF, and JOS would seem to be effective against ureaplasma infections in Tunisian patients. Thus, the early diagnosis and appropriate treatment of *Ureaplasma* spp. may prove to be important in reducing infertility in Tunisia. Furthermore, this study reveals two novel mutations in L22 protein that might be associated with macrolide resistance. Future research are needeed in order to explore the epidemic potential and virulence of *Ureaplasma* serovars that may be associated with the spread of antimicrobial resistance, particularly MDR strains.

## Supplementary information

**Additional file 1: Table S1.** Genes and respective flanking oligonucleotide primers used. **Table S2.** Epidemiologic characteristics of *Ureaplasma* spp. strains used**. Table S3.** In-vitro activity of tetracyclines, fluoroquinolones, and macrolides against 101 human *Ureaplasma* spp. isolates. **Table S4.** Distribution of antimicrobial resistance among patients with genital tract infections and infertility**. Figure S5.** PCR results screening for *tet* (M) (A) and *Int-Tn* genes (B) on 2% agarose gel. **A.** MW: GeneRuler 100 base pairs (bp) DNA Ladder. Lane 1: Negative control. Lane 2–13: Amplicons from the tetracycline-resistant *Ureaplasma* spp. clinical isolates. Lane 14–15: Amplicons from *U. parvum* ATCC 27815 and *U. urealyticum* ATCC 27618. Lane 16–21: Amplicons from tetracycline-sensitive *Ureaplasma* spp. clinical isolates. The *tet* (M) expected gene product is 397 bp based on the primers used. **B.** MW: GeneRuler 100 (bp) DNA Ladder. Lane 1: Negative control. Lane 2: Amplicon from a tetracycline-sensitive *Ureaplasma* spp. clinical isolate. Lane 3–12: Amplicons from tetracycline-resistant *Ureaplasma* spp. clinical isolates. The *Int-Tn* expected gene product is 579 bp based on the primers used. (338 KB).

**Additional file 2: File S1.** Multiple sequence alignment of the 5′-end of *mba* gene of the reference strain UPA1 and 19 *Ureaplasma* serovar 1 clinical strains. **File S2.** Multiple sequence alignment of the 5′-end of *mba* gene of the reference strain UPA3 and 35 *Ureaplasma* serovar 3 clinical strains. **File S3.** Multiple sequence alignment of the 5′-end of *mba* gene of the reference strain UPA6 and 19 *Ureaplasma* serovar 6 clinical strains. **File S4.** Multiple sequence alignment of the 5′-end of *mba* gene of the reference strains UUR4, UUR10, UUR12, UUR13 and 19 *Ureaplasma* serovars 4, 10, 12, 13 clinical strains. **File S5.** Multiple sequence alignment of the 5′-end of *mba* gene of the reference strains UUR2, UUR5, UUR8, UUR9 and 19 *Ureaplasma* serovars 2, 5, 8, 9 clinical strains. **File S6.** Partial sequence alignment of ParC protein of 26 *Ureaplasma* isolates resistant to fluoroquinolones (ParC_consensus correspond to ParC protein of UPA3 and UUR8 reference strains). **File S7.** Partial sequence alignment of ParE protein of 26 *Ureaplasma* isolates resistant to fluoroquinolones (ParE_consensus correspond to ParE protein of UPA3 and UUR8 reference strains). **File S8.** Partial sequence alignment of L22 protein of 26 *Ureaplasma* isolates resistant to macrolides (L22_consensus correspond to L22 protein of UPA3 and UUR8 reference strains). (538.5 KB).

## Data Availability

All data generated or analysed during this study are included in this published article and its additional files.

## Abbreviations

AZM: Azithromycin
CIP: Ciprofloxacin
DOX: Doxycycline
ERY: Erythromycin
JOS: Josamycin
LVX: Levofloxacin
MDR: Multidrug resistance
MIC: Minimum inhibitory concentration
MXF: Moxifloxacin
OFX: Ofloxacin
QRDR: Quinolone resistance determining regions
TET: Tetracycline
UPA: *Ureaplasma parvum*
UUR: *Ureaplasma urealyticum*
