# Supplementary material for: Spread of multidrug resistance among Ureaplasma serovars, Tunisia
Source: Antimicrob Resist Infect Control. 2020 Jan 23;9:19. doi: 10.1186/s13756-020-0681-5 (PMC6979072; doi:10.1186/s13756-020-0681-5)
Supplement: Supplementary file 1 — Additional file 1: Table S1. Genes and respective flanking oligonucleotide primers used. Table S2. Epidemiologic characteristics of Ureaplasma spp. strains used. Table S3. In-vitro activity of tetracyclines, fluoroquinolones, and macrolides against 101 human Ureaplasma spp. isolates. Table S4. Distribution of antimicrobial resistance among patients with genital tract infections and infertility. Figure S5. PCR results screening for tet (M) (A) and Int-Tn genes (B) on 2% agarose gel. A. MW: GeneRuler 100 base pairs (bp) DNA Ladder. Lane 1: Negative control. Lane 2–13: Amplicons from the tetracycline-resistant Ureaplasma spp. clinical isolates. Lane 14–15: Amplicons from U. parvum ATCC 27815 and U. urealyticum ATCC 27618. Lane 16–21: Amplicons from tetracycline-sensitive Ureaplasma spp. clinical isolates. The tet (M) expected gene product is 397 bp based on the primers used. B. MW: GeneRuler 100 (bp) DNA Ladder. Lane 1: Negative control. Lane 2: Amplicon from a tetracycline-sensitive Ureaplasma spp. clinical isolate. Lane 3–12: Amplicons from tetracycline-resistant Ureaplasma spp. clinical isolates. The Int-Tn expected gene product is 579 bp based on the primers used. (338 KB). [file 13756_2020_681_MOESM1_ESM.doc]

**Table S1.** Genes and respective flanking oligonucleotide primers used.

| **Target gene** | **Primer** | **Sequence (5’ to 3’)** | **Reference** |
| --- | --- | --- | --- |
| *mba* (403/448 bp) | UMS-125  UMA226 | GTATTTGCAATCTTTATATGTTTTCG  CAGCTGATGTAAGTGCAGCATTAAATTC | [19] |
| *tet*(M) (397 bp) | TetMF  TetMR | TTATCAACGGTTTATCAGG  CGTATATATGCAAGACG | [16] |
| *Int-Tn* (579 bp) | int1  int2 | TGACACTCTGCCAGCTTAC  CCATAGGAACTTGACGTTGG | [16] |
| *gyrA* (336 bp) | *gyrA-1*  *gyrA-2* | 5TTGCTGCTTTCGAAAACGG  CTGATGGTAAAACACTTGG | [23] |
| *gyrB* (310 bp) | *gyrB-3*  *gyrB-4* | CCTGGTAAATTAGCTGACTG  TTCGAATATGACTGCCATC | [23] |
| *parC* (309 bp) | *parC-5*  *parC-6* | ACGCAATGAGTGAATTAGG  CACTATCATCAAAGTTTGGAC | [23] |
| *parE* (313 bp) | *parE-7*  *parE-8* | ATGGGCGGAAAATTAACGC  CTTGGATGTGACTACCATCG | [23] |
| 23S rRNA domain II (330 bp) | UP23S-30  UP23S-31 | TGCCTTTTGAAGTATGAGCC  TGGCGCCATCATAGATTCAG | [24] |
| 23S rRNA domain V (352 bp) | MH23S-11  MP23S-22 | TAACTATAACGGTCCTAAGG  GGCGACCGCCCCAGTCAAAC | [24] |
| L4 (392 bp) | UPL4-U  UPL4-R | TCTATTGATGGTAACTTCGC  GTTGAAGGTGTTTCTAAATCGC | [24] |
| L22 (458 bp) | UPL22U  UPL22-R | TTCGCACCGTAAAGCTTCTC  GTTCTGGATCAACGTTTTCG | [24] |

**Table S2.** Epidemiologic characteristics of *Ureaplasma* spp. strains used.

| Strain name | Year of isolation | Source | Sex | Clinical manifestations |  |
| --- | --- | --- | --- | --- | --- |
| UPA3 ATCC 27815 | 1967 | Urethral exudate | Male | Nongonococcal urethritis |  |
| UUR8 ATCC 27618 | 1961 | Urethral exudate | Male | Nongonococcal urethritis |  |
| U1 | 2005 | Vaginal swab | Female | Gynecological infections |  |
| U2 | 2005 | Vaginal swab | Female | Gynecological infections |  |
| U3 | 2005 | Vaginal swab | Female | Infertility |  |
| U4 | 2006 | Vaginal swab | Female | Infertility |  |
| U5 | 2006 | Sperme | Male | Infertility |  |
| U6 | 2006 | Vaginal swab | Female | Gynecological infections |  |
| U7 | 2006 | Sperme | Male | Gynecological infections |  |
| U8 | 2007 | Sperme | Male | Infertility |  |
| U9 | 2007 | Vaginal swab | Female | Infertility |  |
| U10 | 2007 | Sperme | Male | Infertility |  |
| U11 | 2007 | Vaginal swab | Female | Infertility |  |
| U12 | 2008 | Sperme | Male | Gynecological infections |  |
| U13 | 2008 | Vaginal swab | Female | Infertility |  |
| U14 | 2008 | Vaginal swab | Female | Gynecological infections |  |
| U15 | 2008 | Sperme | Male | Infertility |  |
| U16 | 2009 | Sperme | Male | Infertility |  |
| U17 | 2009 | Vaginal swab | Female | Infertility |  |
| U18 | 2009 | Vaginal swab | Female | Infertility |  |
| U19 | 2009 | Vaginal swab | Female | Infertility |  |
| U20 | 2009 | Vaginal swab | Female | Infertility |  |
| U21 | 2009 | Vaginal swab | Female | Infertility |  |
| U22 | 2010 | Vaginal swab | Female | Infertility |  |
| U23 | 2010 | Vaginal swab | Female | Infertility |  |
| U24 | 2010 | Vaginal swab | Female | Infertility |  |
| U25 | 2010 | Vaginal swab | Female | Infertility |  |
| U26 | 2010 | Vaginal swab | Female | Infertility |  |
| U27 | 2011 | Vaginal swab | Female | Infertility |  |
| U28 | 2011 | Vaginal swab | Female | Gynecological infections |  |
| U29 | 2011 | Vaginal swab | Female | Infertility |  |
| U30 | 2011 | Vaginal swab | Female | Infertility |  |
| U31 | 2011 | Vaginal swab | Female | Infertility |  |
| U32 | 2011 | Vaginal swab | Female | Gynecological infections |  |
| U33 | 2012 | Vaginal swab | Female | Infertility |  |
| U34 | 2012 | Sperme | Male | Infertility |  |
| U35 | 2012 | Vaginal swab | Female | Infertility |  |
| U36 | 2012 | Vaginal swab | Female | Infertility |  |
| U37 | 2012 | Sperme | Male | Infertility |  |
| U38 | 2012 | Vaginal swab | Female | Infertility |  |
| U39 | 2012 | Sperme | Male | Infertility |  |
| U40 | 2012 | Vaginal swab | Female | Infertility |  |
| U41 | 2012 | Vaginal swab | Female | Infertility |  |
| U42 | 2013 | Vaginal swab | Female | Infertility |  |
| U43 | 2013 | Vaginal swab | Female | Infertility |  |
| U44 | 2013 | Sperme | Male | Infertility |  |
| U45 | 2013 | Vaginal swab | Female | Infertility |  |
| U46 | 2013 | Vaginal swab | Female | Gynecological infections |  |
| U47 | 2013 | Vaginal swab | Female | Infertility |  |
| U48 | 2013 | Vaginal swab | Female | Infertility |  |
| U49 | 2013 | Vaginal swab | Female | Infertility |  |
| U50 | 2013 | Vaginal swab | Female | Infertility |  |
| U51 | 2013 | Vaginal swab | Female | Infertility |  |
| U52 | 2014 | Vaginal swab | Female | Infertility |  |
| U53 | 2014 | Vaginal swab | Female | Gynecological infections |  |
| U54 | 2014 | Vaginal swab | Female | Infertility |  |
| U55 | 2014 | Sperme | Male | Infertility |  |

| U56 | 2014 | Vaginal swab | Female | Infertility |
| --- | --- | --- | --- | --- |
| U57 | 2014 | Sperme | Male | Infertility |
| U58 | 2014 | Vaginal swab | Female | Gynecological infections |
| U59 | 2014 | Vaginal swab | Female | Gynecological infections |
| U60 | 2014 | Vaginal swab | Female | Infertility |
| U61 | 2015 | Vaginal swab | Female | Infertility |
| U62 | 2015 | Sperme | Male | Infertility |
| U63 | 2015 | Sperme | Male | Infertility |
| U64 | 2015 | Sperme | Male | Infertility |
| U65 | 2015 | Vaginal swab | Female | Gynecological infections |
| U66 | 2015 | Sperme | Male | Infertility |
| U67 | 2015 | Vaginal swab | Female | Infertility |
| U68 | 2015 | Vaginal swab | Female | Infertility |
| U69 | 2015 | Vaginal swab | Female | Gynecological infections |
| U70 | 2015 | Vaginal swab | Female | Infertility |
| U71 | 2015 | Vaginal swab | Female | Infertility |
| U72 | 2015 | Vaginal swab | Female | Infertility |
| U73 | 2015 | Vaginal swab | Female | Infertility |
| U74 | 2016 | Vaginal swab | Female | Infertility |
| U75 | 2016 | Vaginal swab | Female | Infertility |
| U76 | 2016 | Vaginal swab | Female | Infertility |
| U77 | 2016 | Vaginal swab | Female | Infertility |
| U78 | 2016 | Sperme | Male | Infertility |
| U79 | 2016 | Vaginal swab | Female | Infertility |
| U80 | 2016 | Sperme | Male | Infertility |
| U81 | 2016 | Vaginal swab | Female | Infertility |
| U82 | 2016 | Sperme | Male | Infertility |
| U83 | 2016 | Vaginal swab | Female | Infertility |
| U84 | 2016 | Vaginal swab | Female | Infertility |
| U85 | 2016 | Sperme | Male | Infertility |
| U86 | 2017 | Vaginal swab | Female | Infertility |
| U87 | 2017 | Vaginal swab | Female | Gynecological infections |
| U88 | 2017 | Sperme | Male | Infertility |
| U89 | 2017 | Sperme | Male | Gynecological infections |
| U90 | 2017 | Sperme | Male | Infertility |
| U91 | 2017 | Vaginal swab | Female | Infertility |
| U92 | 2017 | Sperme | Male | Infertility |
| U93 | 2017 | Vaginal swab | Female | Infertility |
| U94 | 2017 | Vaginal swab | Female | Infertility |
| U95 | 2017 | Vaginal swab | Female | Infertility |
| U96 | 2017 | Vaginal swab | Female | Infertility |
| U97 | 2017 | Vaginal swab | Female | Infertility |
| U98 | 2017 | Vaginal swab | Female | Infertility |
| U99 | 2017 | Vaginal swab | Female | Infertility |
| U100 | 2017 | Vaginal swab | Female | Infertility |
| U101 | 2017 | Vaginal swab | Female | Infertility |

**Table S3.** *In-vitro* activity of tetracyclines, fluoroquinolones, and macrolides against 101 human *Ureaplasma* spp. isolates.

| Strain | Serovar |  |  |  |  | MIC (mg/L) |  |  |  |  |
| --- | --- | --- | --- | --- | --- | --- | --- | --- | --- | --- |
| name |  |  |  |  |  |  |  |  |  |  |
|  |  | TET | DOX | OFX | CIP | LVX | MXF | AZM | ERY | JOS |
|  |  |  |  |  |  |  |  |  |  |  |
| ATCC | UPA3 | 0.06 | 0.03 | 0.25 | 0.5 | 0.125 | 0.06 | 0.06 | 2 | 0.03 |
| 27815 |  |  |  |  |  |  |  |  |  |  |
| ATCC | UUR8 | 0.06 | 0.03 | 0.5 | 0.5 | 0.25 | 0.06 | 0.06 | 2 | 0.03 |
| 27618 |  |  |  |  |  |  |  |  |  |  |
| U1 | UPA6 | 0.25 | 0.03 | 2 | 8 | 1 | 0.5 | 1 | 64 | 0.5 |
| U2 | UPA1 | 2 | 0.125 | 2 | 4 | 1 | 0.5 | 0.5 | 16 | 0.5 |
| U3 | UPA6 | 0.5 | 0.03 | 1 | 4 | 1 | 0.5 | 2 | 64 | 0.5 |
| U4 | UUR4, 10, 12, 13 | 1 | 0.03 | 2 | 4 | 2 | 0.5 | 1 | 64 | 0.03 |
| U5 | UUR4, 10, 12, 13 | 1 | 0.03 | 2 | 4 | 2 | 0.5 | 1 | 64 | 0.03 |
| U6 | UPA3 | 0.5 | 0.125 | 4 | 4 | 0.25 | 0.5 | 0.25 | 16 | 0.125 |
| U7 | UPA3 | 2 | 0.125 | 2 | 4 | 0.5 | 1 | 1 | 64 | 0.5 |
| U8 | UPA3 | 2 | 0.125 | 2 | 4 | 0.25 | 1 | 0.5 | 64 | 0.5 |
| U9 | UPA3 | 0.06 | 0.03 | 1 | 4 | 0.5 | 0.5 | 1 | 64 | 0.5 |
| U10 | UPA6 | 0.25 | 0.03 | 4 | 8 | 1 | 0.5 | 0.5 | 16 | 0.5 |
| U11 | UUR4, 10, 12, 13 | 1 | 0.125 | 4 | 4 | 4 | 1 | 1 | 32 | 0.03 |
| U12 | UPA1 | 1 | 0.25 | 4 | 8 | 1 | 1 | 2 | 64 | 0.5 |
| U13 | UUR4, 10, 12, 13 | 1 | 0.25 | 4 | 8 | 4 | 0.5 | 0.25 | 64 | 0.03 |
| U14 | UPA1 | 1 | 0.125 | 4 | 16 | 1 | 0.5 | 2 | 64 | 0.5 |
| U15 | UPA3 | 1 | 0.125 | 4 | 8 | 0.5 | 0.5 | 1 | 16 | 0.5 |
| U16 | UPA1 | 2 | 0.125 | 1 | 4 | 0.5 | 0.25 | 0.5 | 16 | 0.125 |
| U17 | UPA3 | 2 | 1 | 2 | 4 | 0.5 | 1 | 1 | 16 | 0.5 |
| U18 | UPA1 | 2 | 0.125 | 1 | 4 | 0.5 | 0.25 | 0.5 | 16 | 0.125 |
| U19 | UPA6 | 0.5 | 0.125 | 0.5 | 4 | 0.5 | 0.125 | 0.25 | 32 | 0.125 |
| U20 | UPA3 | 1 | 0.125 | 2 | 4 | 0.5 | 0.5 | 2 | 64 | 0.5 |
| U21 | UUR2, 5, 8, 9 | 8 | 0.5 | 8 | 8 | 4 | 2 | 1 | 64 | 0.25 |
| U22 | UUR2, 5, 8, 9 | 2 | 0.125 | 4 | 4 | 4 | 1 | 2 | 16 | 0.5 |
| U23 | UPA3 | 2 | 1 | 2 | 4 | 0.5 | 1 | 1 | 16 | 0.5 |
| U24 | UUR4, 10, 12, 13 | 2 | 0.5 | 4 | 4 | 4 | 0.5 | 1 | 64 | 0.03 |
| U25 | UPA3 | 0.5 | 0.125 | 4 | 4 | 0.25 | 0.5 | 0.25 | 16 | 0.125 |
| U26 | UPA3 | 1 | 0.125 | 1 | 4 | 0.5 | 1 | 1 | 64 | 0.5 |
| U27 | UPA3 | 2 | 1 | 2 | 4 | 1 | 0.5 | 1 | 16 | 0.5 |
| U28 | UPA1 | 1 | 0.125 | 2 | 4 | 1 | 0.125 | 1 | 16 | 0.25 |
| U29 | UUR4, 10, 12, 13 | 2 | 0.125 | 4 | 8 | 1 | 0.5 | 1 | 16 | 1 |
| U30 | UUR4, 10, 12, 13 | 1 | 0.03 | 4 | 8 | 4 | 0.5 | 1 | 32 | 0.03 |
| U31 | UPA3 | 4 | 0.25 | 4 | 8 | 1 | 0.5 | 1 | 32 | 1 |
| U32 | UPA1 | 1 | 0.125 | 2 | 4 | 1 | 0.125 | 1 | 16 | 0.25 |
| U33 | UUR2, 5, 8, 9 | 16 | 1 | 4 | 4 | 4 | 0.5 | 1 | 64 | 1 |
| U34 | UUR2, 5, 8, 9 | 2 | 0.5 | 4 | 8 | 4 | 0.5 | 0.5 | 16 | 1 |
| U35 | UPA6 | 1 | 0.125 | 1 | 4 | 1 | 0.25 | 0.25 | 32 | 0.125 |
| U36 | UUR4, 10, 12, 13 | 1 | 0.25 | 4 | 8 | 4 | 0.5 | 0.25 | 64 | 0.03 |
| U37 | UPA3 | 1 | 0.125 | 2 | 4 | 1 | 0.5 | 0.5 | 16 | 0.5 |
| U38 | UPA1 | 1 | 0.25 | 4 | 8 | 1 | 0.5 | 2 | 64 | 0.5 |

| U39 | UPA1 | 1 | 0.25 | 4 | 8 | 1 | 0.5 | 2 | 64 | 0.5 |
| --- | --- | --- | --- | --- | --- | --- | --- | --- | --- | --- |
| U40 | UUR2, 5, 8, 9 | 0.125 | 0.06 | 4 | 4 | 4 | 0.5 | 0.5 | 64 | 0.5 |
| U41 | UPA6 | 0.5 | 0.125 | 1 | 4 | 1 | 0.25 | 0.5 | 32 | 0.125 |
| U42 | UUR4, 10, 12, 13 | 4 | 0.25 | 4 | 4 | 2 | 0.5 | 1 | 32 | 0.5 |
| U43 | UPA6 | 0.5 | 0.125 | 1 | 4 | 1 | 0.25 | 0.5 | 32 | 0.125 |
| U44 | UPA3 | 0.5 | 0.06 | 2 | 4 | 1 | 0.5 | 2 | 32 | 0.5 |
| U45 | UUR4, 10, 12, 13 | 2 | 0.125 | 4 | 8 | 1 | 0.5 | 1 | 16 | 1 |
| U46 | UPA3 | 1 | <0.03 | 2 | 4 | 1 | 0.5 | 2 | 32 | 0.5 |
| U47 | UUR4, 10, 12, 13 | 4 | 0.125 | 4 | 4 | 4 | 1 | 0.5 | 16 | 0.5 |
| U48 | UUR4, 10, 12, 13 | 1 | 0.125 | 4 | 4 | 2 | 0.5 | 0.5 | 32 | 0.25 |
| U49 | UPA3 | 1 | 0.125 | 2 | 4 | 0.25 | 0.25 | 1 | 16 | 0.25 |
| U50 | UPA3 | 2 | 0.125 | 2 | 4 | 0.5 | 0.5 | 2 | 16 | 0.5 |
| U51 | UPA3 | 2 | 0.125 | 2 | 4 | 0.5 | 0.5 | 2 | 32 | 0.5 |
| U52 | UUR2, 5, 8, 9 | 2 | 1 | 4 | 8 | 4 | 0.5 | 1 | 64 | 0.5 |
| U53 | UPA3 | 1 | 0.03 | 1 | 4 | 0.03 | 0.125 | 1 | 32 | 0.5 |
| U54 | UUR4, 10, 12, 13 | 4 | 0.25 | 4 | 8 | 4 | 1 | 2 | 64 | 0.5 |
| U55 | UUR2, 5, 8, 9 | 16 | 0.5 | 8 | 8 | 4 | 2 | 2 | 64 | 0.5 |
| U56 | UUR4, 10, 12, 13 | 2 | 0.06 | 4 | 4 | 4 | 1 | 2 | 64 | 0.25 |
| U57 | UPA3 | 2 | 0.125 | 4 | 4 | 0.125 | 1 | 1 | 16 | 0.5 |
| U58 | UPA1 | 0.5 | 0.125 | 4 | 4 | 1 | 1 | 1 | 32 | 0.5 |
| U59 | UPA1 | 0.5 | 0.125 | 4 | 4 | 1 | 1 | 1 | 32 | 0.5 |
| U60 | UPA1 | 1 | 0.125 | 2 | 4 | 1 | 0.5 | 0.5 | 16 | 0.25 |
| U61 | UUR2, 5, 8, 9 | 1 | 0.125 | 4 | 4 | 2 | 1 | 1 | 64 | 0.03 |
| U62 | UPA3 | 2 | 0.25 | 2 | 4 | 0.5 | 1 | 0.5 | 16 | 0.5 |
| U63 | UPA6 | 1 | 0.125 | 1 | 4 | 1 | 0.25 | 0.5 | 32 | 0.125 |
| U64 | UPA3 | 2 | 0.125 | 4 | 4 | 0.125 | 1 | 1 | 16 | 0.5 |
| U65 | UPA1 | 1 | 0.125 | 2 | 4 | 1 | 0.5 | 0.25 | 16 | 0.125 |
| U66 | UUR4, 10, 12, 13 | 4 | 0.125 | 4 | 4 | 4 | 1 | 0.5 | 16 | 0.5 |
| U67 | UUR4, 10, 12, 13 | 2 | 0.06 | 4 | 4 | 4 | 1 | 2 | 64 | 0.25 |
| U68 | UPA1 | 1 | 0.125 | 2 | 4 | 1 | 0.5 | 0.25 | 16 | 0.125 |
| U69 | UPA3 | 1 | 0.125 | 2 | 4 | 0.5 | 0.5 | 1 | 32 | 0.25 |
| U70 | UPA3 | 4 | 0.25 | 1 | 4 | 0.25 | 0.125 | 0.25 | 16 | <0.03 |
| U71 | UPA3 | 16 | 0.25 | 1 | 4 | 0.5 | 0.5 | 0.25 | 16 | 0.25 |
| U72 | UPA3 | 2 | 0.125 | 4 | 4 | 1 | 1 | 1 | 16 | 0.5 |
| U73 | UPA3 | 1 | 0.06 | 2 | 4 | 0.5 | 0.5 | 2 | 32 | 0.25 |
| U74 | UUR4, 10, 12, 13 | 2 | 0.125 | 4 | 8 | 2 | 0.5 | 0.5 | 16 | 0.5 |
| U75 | UPA3 | 32 | 0.25 | 2 | 4 | 1 | 0.5 | 1 | 16 | 0.5 |
| U76 | UPA1 | 1 | 0.125 | 2 | 4 | 1 | 0.5 | 0.25 | 16 | 0.125 |
| U77 | UPA3 | 0.5 | 0.06 | 2 | 4 | 1 | 1 | 0.25 | 16 | 0.25 |
| U78 | UPA3 | 1 | 0.25 | 1 | 4 | 0.25 | 0.25 | 0.5 | 16 | 0.25 |
| U79 | UPA3 | 0.25 | 0.06 | 1 | 4 | 0.5 | 0.5 | 0.5 | 16 | 0.125 |
| U80 | UPA6 | 2 | 0.25 | 2 | 4 | 1 | 0.5 | 1 | 16 | 0.5 |
| U81 | UPA6 | 0.5 | 0.125 | 1 | 4 | 1 | 0.25 | 0.25 | 32 | 0.125 |
| U82 | UPA1 | 0.5 | 0.125 | 1 | 4 | 1 | 0.25 | 0.5 | 16 | 0.125 |
| U83 | UPA6 | 1 | 0.125 | 2 | 4 | 2 | 0.25 | 1 | 32 | 0.125 |
| U84 | UUR2, 5, 8, 9 | 8 | 0.25 | 8 | 8 | 4 | 2 | 1 | 64 | 0.5 |

| U85 | UPA6 | 1 | 0.125 | 1 | 4 | 1 | 0.25 | 0.5 | 64 | 0.125 |
| --- | --- | --- | --- | --- | --- | --- | --- | --- | --- | --- |
| U86 | UPA6 | 1 | 0.125 | 1 | 4 | 1 | 0.25 | 0.5 | 32 | 0.25 |
| U87 | UPA3 | 2 | 0.06 | 4 | 4 | 1 | 1 | 1 | 16 | 0.5 |
| U88 | UPA3 | 0.5 | 0.125 | 2 | 4 | 2 | 0.5 | 0.5 | 16 | 0.125 |
| U89 | UPA6 | 1 | 0.125 | 2 | 4 | 2 | 0.25 | 1 | 16 | 0.5 |
| U90 | UUR4, 10, 12, 13 | 0.25 | 0.06 | 2 | 4 | 1 | 0.25 | 0.5 | 32 | 0.5 |
| U91 | UPA6 | 0.5 | 0.06 | 2 | 8 | 2 | 0.25 | 0.5 | 16 | 0.125 |
| U92 | UPA6 | 0.25 | 0.06 | 2 | 8 | 2 | 0.25 | 0.5 | 64 | 0.125 |
| U93 | UPA6 | 0.5 | 0.06 | 2 | 8 | 2 | 0.25 | 1 | 16 | 0.5 |
| U94 | UPA1 | 0.5 | 0.06 | 1 | 4 | 1 | 0.25 | 1 | 16 | 0.5 |
| U95 | UUR4, 10, 12, 13 | 0.5 | 0.06 | 1 | 4 | 1 | 0.25 | 1 | 16 | 0.5 |
| U96 | UPA1 | 1 | 0.06 | 1 | 4 | 1 | 0.25 | 1 | 16 | 0.5 |
| U97 | UPA1 | 0.25 | 0.06 | 0.5 | 4 | 0.5 | 0.25 | 1 | 16 | 0.5 |
| U98 | UPA3 | 1 | 0.25 | 1 | 4 | 1 | 0.125 | 1 | 32 | 0.5 |
| U99 | UPA6 | 0.5 | 0.5 | 1 | 4 | 1 | 0.25 | 0.5 | 32 | 0.5 |
| U100 | UPA6 | 0.5 | 0.06 | 1 | 4 | 0.5 | 0.25 | 0.5 | 16 | 0.25 |
| U101 | UPA3 | 16 | 1 | 1 | 4 | 2 | 0.25 | 0.5 | 16 | 0.25 |
|  |  |  |  |  |  |  |  |  |  |  |

**Table S4.** Distribution of antimicrobial resistance among patients with genital tract infections and infertility.

| Antibiotic  Clinical  status | TET (*P* < 0.0004) | | OFX (*P* < 0.0001) | | | LVX (*P* = 0.2343) | |
| --- | --- | --- | --- | --- | --- | --- | --- |
|  | S | R | S | MS | R | S | R |
| Genital infections | 81.25% | 18.75% | 6.25% | 50% | 43.75% | 87.50% | 12.50% |
| Infertility | 58.82% | 41.18% | 30.59% | 31.76% | 37.65% | 81.18% | 18.82% |

S: sensitive; R: resistant; MS: moderately sensitive.


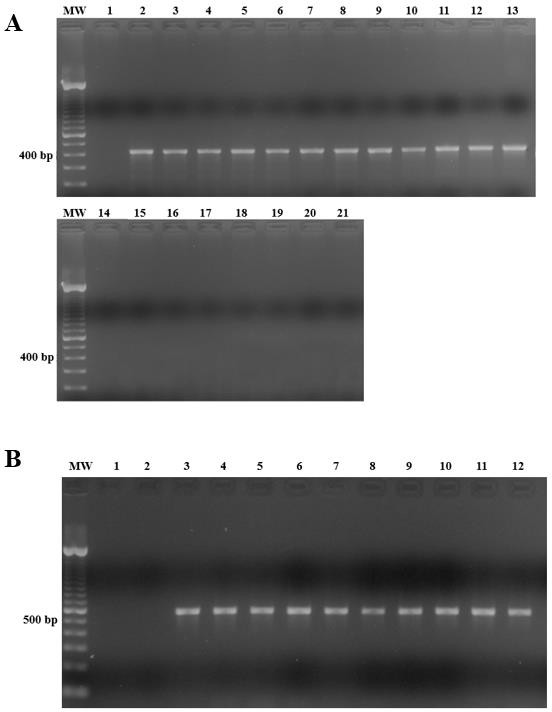


**Figure S5.** PCR results screening for *tet*(M) (A) and *Int-Tn* genes (B) on 2% agarose gel.

**A.** MW: GeneRuler 100 base pairs (bp) DNA Ladder. Lane 1: Negative control. Lane 2-13: Amplicons from the tetracycline-resistant *Ureaplasma* spp. clinical isolates. Lane 14-15: Amplicons from *U. parvum* ATCC 27815 and *U. urealyticum* ATCC 27618. Lane 16-21: Amplicons from tetracycline-sensitive *Ureaplasma* spp. clinical isolates. The *tet*(M) expected gene product is 397 bp based on the primers used.

**B.** MW: GeneRuler 100 (bp) DNA Ladder. Lane 1: Negative control. Lane 2: Amplicon from a tetracycline-sensitive *Ureaplasma* spp. clinical isolate. Lane 3-12: Amplicons from tetracycline-resistant *Ureaplasma* spp. clinical isolates. The *Int-Tn* expected gene product is 579 bp based on the primers used.
