# Supplementary material for: Spread of multidrug resistance among Ureaplasma serovars, Tunisia
Source: Antimicrob Resist Infect Control. 2020 Jan 23;9:19. doi: 10.1186/s13756-020-0681-5 (PMC6979072; doi:10.1186/s13756-020-0681-5)
Supplement: Supplementary file 2 — Additional file 2: File S1. Multiple sequence alignment of the 5′-end of mba gene of the reference strain UPA1 and 19 Ureaplasma serovar 1 clinical strains. File S2. Multiple sequence alignment of the 5′-end of mba gene of the reference strain UPA3 and 35 Ureaplasma serovar 3 clinical strains. File S3. Multiple sequence alignment of the 5′-end of mba gene of the reference strain UPA6 and 19 Ureaplasma serovar 6 clinical strains. File S4. Multiple sequence alignment of the 5′-end of mba gene of the reference strains UUR4, UUR10, UUR12, UUR13 and 19 Ureaplasma serovars 4, 10, 12, 13 clinical strains. File S5. Multiple sequence alignment of the 5′-end of mba gene of the reference strains UUR2, UUR5, UUR8, UUR9 and 19 Ureaplasma serovars 2, 5, 8, 9 clinical strains. File S6. Partial sequence alignment of ParC protein of 26 Ureaplasma isolates resistant to fluoroquinolones (ParC_consensus correspond to ParC protein of UPA3 and UUR8 reference strains). File S7. Partial sequence alignment of ParE protein of 26 Ureaplasma isolates resistant to fluoroquinolones (ParE_consensus correspond to ParE protein of UPA3 and UUR8 reference strains). File S8. Partial sequence alignment of L22 protein of 26 Ureaplasma isolates resistant to macrolides (L22_consensus correspond to L22 protein of UPA3 and UUR8 reference strains). (538.5 KB). [file 13756_2020_681_MOESM2_ESM.doc]

10 20 30 40 50 60 70 80 90 100 110 120

....|....|....|....|....|....|....|....|....|....|....|....|....|....|....|....|....|....|....|....|....|....|....|....|

**UPA1** **GTATTTGCAATCTTTATATGTTTTCGTTAAAATTAAAAATTAATTACTGTAGAAATTATGTAAGATTGCTAAATCTTAGTGTTCATATTTTTTACACATATTAAATAAAGACAATAAAAT**

**U2** **........................................................................................................................**

**U12** **........................................................................................................................**

**U14** **........................................................................................................................**

**U16** **........................................................................................................................**

**U18** **........................................................................................................................**

**U28** **........................................................................................................................**

**U32** **........................................................................................................................**

**U38** **........................................................................................................................**

**U39** **........................................................................................................................**

**U58** **........................................................................................................................**

**U59** **........................................................................................................................**

**U60** **........................................................................................................................**

**U65** **........................................................................................................................**

**U68** **........................................................................................................................**

**U76** **........................................................................................................................**

**U82** **........................................................................................................................**

**U94** **........................................................................................................................**

**U96** **........................................................................................................................**

**U97** **........................................................................................................................**

130 140 150 160 170 180 190 200 210 220 230 240

....|....|....|....|....|....|....|....|....|....|....|....|....|....|....|....|....|....|....|....|....|....|....|....|

**UPA1** **GACATATTTTTTATATTAGGAGAATCATAAATGAAATTATTAAAAAATAAAAAATTCTGAGCTATGACATTAGGAGTTACCTTAGTTGGAGCTGGAATAGTTGCTATAGCAGCTTCATGT**

**U2** **........................................................................................................................**

**U12** **........................................................................................................................**

**U14** **........................................................................................................................**

**U16** **........................................................................................................................**

**U18** **........................................................................................................................**

**U28** **........................................................................................................................**

**U32** **........................................................................................................................**

**U38** **........................................................................................................................**

**U39** **........................................................................................................................**

**U58** **........................................................................................................................**

**U59** **........................................................................................................................**

**U60** **........................................................................................................................**

**U65** **........................................................................................................................**

**U68** **........................................................................................................................**

**U76** **........................................................................................................................**

**U82** **........................................................................................................................**

**U94** **........................................................................................................................**

**U96** **........................................................................................................................**

**U97** **........................................................................................................................**

250 260 270 280 290 300 310 320 330 340 350 360

....|....|....|....|....|....|....|....|....|....|....|....|....|....|....|....|....|....|....|....|....|....|....|....|

**UPA1** **TCTAATTCAACCGTTAAATCTAAATTAAGTAACCAATTTGCTAAATCAACAGACGATAAAAGTTTTTATGCGGTTTACGAAATTGAAAACTTTAAAGATCTAAGTGATAATGATAAAAAA**

**U2** **........................................................................................................................**

**U12** **........................................................................................................................**

**U14** **........................................................................................................................**

**U16** **........................................................................................................................**

**U18** **........................................................................................................................**

**U28** **........................................................................................................................**

**U32** **........................................................................................................................**

**U38** **........................................................................................................................**

**U39** **........................................................................................................................**

**U58** **........................................................................................................................**

**U59** **........................................................................................................................**

**U60** **........................................................................................................................**

**U65** **........................................................................................................................**

**U68** **........................................................................................................................**

**U76** **........................................................................................................................**

**U82** **........................................................................................................................**

**U94** **........................................................................................................................**

**U96** **........................................................................................................................**

**U97** **........................................................................................................................**

370 380 390 400

_....|....|....|....|....|....|....|....|.._.

**UPA1** **TCATTAAATGACATTGAATTTAATGCTGCACTTACATCAGCTG**

**U2** **...........................................**

**U12** **...........................................**

**U14** **...........................................**

**U16** **...........................................**

**U18** **...........................................**

**U28** **...........................................**

**U32** **...........................................**

**U38** **...........................................**

**U39** **...........................................**

**U58** **...........................................**

**U59** **...........................................**

**U60** **...........................................**

**U65** **...........................................**

**U68** **...........................................**

**U76** **...........................................**

**U82** **...........................................**

**U94** **...........................................**

**U96** **...........................................**

**U97** **...........................................**

**File S1.** Multiple sequence alignment of the 5’-end of *mba* gene of the reference strain UPA1 and 19 *Ureaplasma* serovar 1 clinical strains.

10 20 30 40 50 60 70 80 90 100 110 120

....|....|....|....|....|....|....|....|....|....|....|....|....|....|....|....|....|....|....|....|....|....|....|....|

**UPA3** **GTATTTGCAATCTTTATATGTTTTCGTTAAAATTAAAAATTAATTACTGTAGAAATTATGTAAGATTACCAAATCTTAGTGTTCATATTTTTTACATATATTAAATAAAAACAATAAAAT**

**U6**  **........................................................................................................................**

**U7**  **........................................................................................................................**

**U8**  **........................................................................................................................**

**U9**  **........................................................................................................................**

**U15**  **........................................................................................................................**

**U17**  **........................................................................................................................**

**U20**  **........................................................................................................................**

**U23**  **........................................................................................................................**

**U25**  **........................................................................................................................**

**U26**  **........................................................................................................................**

**U27**  **........................................................................................................................**

**U31**  **........................................................................................................................**

**U37**  **........................................................................................................................**

**U44**  **........................................................................................................................**

**U46**  **........................................................................................................................**

**U49**  **........................................................................................................................**

**U50**  **........................................................................................................................**

**U51**  **........................................................................................................................**

**U53**  **........................................................................................................................**

**U57**  **........................................................................................................................**

**U62**  **........................................................................................................................**

**U64**  **........................................................................................................................**

**U69**  **........................................................................................................................**

**U70**  **........................................................................................................................**

**U71**  **........................................................................................................................**

**U72**  **........................................................................................................................**

**U73**  **........................................................................................................................**

**U75**  **........................................................................................................................**

**U77**  **........................................................................................................................**

**U78**  **........................................................................................................................**

**U79**  **........................................................................................................................**

**U87**  **........................................................................................................................**

**U88**  **........................................................................................................................**

**U98**  **........................................................................................................................**

**U101** **........................................................................................................................**

130 140 150 160 170 180 190 200 210 220 230 240

....|....|....|....|....|....|....|....|....|....|....|....|....|....|....|....|....|....|....|....|....|....|....|....|

**UPA3** **GACATATTTTTTATATTAGGAGAATCATAAATGAAATTATTAAAAAATAAAAAATTCTGAGCTATGACATTAGGTGTTACCTTAGTTGGAGCTGGAATAGTTGCTATAGCAGCTTCATGT**

**U6**  **........................................................................................................................**

**U7**  **........................................................................................................................**

**U8**  **........................................................................................................................**

**U9**  **........................................................................................................................**

**U15**  **........................................................................................................................**

**U17**  **........................................................................................................................**

**U20**  **........................................................................................................................**

**U23**  **........................................................................................................................**

**U25**  **........................................................................................................................**

**U26**  **........................................................................................................................**

**U27**  **........................................................................................................................**

**U31**  **........................................................................................................................**

**U37**  **........................................................................................................................**

**U44**  **........................................................................................................................**

**U46**  **........................................................................................................................**

**U49**  **........................................................................................................................**

**U50**  **........................................................................................................................**

**U51**  **........................................................................................................................**

**U53**  **........................................................................................................................**

**U57**  **........................................................................................................................**

**U62**  **........................................................................................................................**

**U64**  **........................................................................................................................**

**U69**  **........................................................................................................................**

**U70**  **........................................................................................................................**

**U71**  **........................................................................................................................**

**U72**  **........................................................................................................................**

**U73**  **........................................................................................................................**

**U75**  **........................................................................................................................**

**U77**  **........................................................................................................................**

**U78**  **........................................................................................................................**

**U79**  **........................................................................................................................**

**U87**  **........................................................................................................................**

**U88**  **........................................................................................................................**

**U98**  **........................................................................................................................**

**U101** **........................................................................................................................**

250 260 270 280 290 300 310 320 330 340 350 360

....|....|....|....|....|....|....|....|....|....|....|....|....|....|....|....|....|....|....|....|....|....|....|....|

**UPA3** **TCTAATTCAACTGTTAAATCTAAGTTAAGTAACCAATTTGCTAAATCAACAGACGGTAAAAGTTTTTATGCGGTTTACGAAATTGAAAACTTTAAAGATCTAAGTAATGATGATAAAAAA**

**U6**  **........................................................................................................................**

**U7**  **........................................................................................................................**

**U8**  **........................................................................................................................**

**U9**  **........................................................................................................................**

**U15**  **........................................................................................................................**

**U17**  **........................................................................................................................**

**U20**  **........................................................................................................................**

**U23**  **........................................................................................................................**

**U25**  **........................................................................................................................**

**U26**  **........................................................................................................................**

**U27**  **........................................................................................................................**

**U31**  **........................................................................................................................**

**U37**  **........................................................................................................................**

**U44**  **........................................................................................................................**

**U46**  **........................................................................................................................**

**U49**  **........................................................................................................................**

**U50**  **........................................................................................................................**

**U51**  **........................................................................................................................**

**U53**  **........................................................................................................................**

**U57**  **........................................................................................................................**

**U62**  **........................................................................................................................**

**U64**  **........................................................................................................................**

**U69**  **........................................................................................................................**

**U70**  **........................................................................................................................**

**U71**  **........................................................................................................................**

**U72**  **........................................................................................................................**

**U73**  **........................................................................................................................**

**U75**  **........................................................................................................................**

**U77**  **........................................................................................................................**

**U78**  **........................................................................................................................**

**U79**  **........................................................................................................................**

**U87**  **........................................................................................................................**

**U88**  **........................................................................................................................**

**U98**  **........................................................................................................................**

**U101** **........................................................................................................................**

370 380 390 400

....|....|....|....|....|....|....|....|...

**UPA3** **TCATTAAGTAACATTGAATTTAATGCTGCACTTACATCAGCTG**

**U6**  **...........................................**

**U7**  **...........................................**

**U8**  **...........................................**

**U9**  **...........................................**

**U15**  **...........................................**

**U17**  **...........................................**

**U20**  **...........................................**

**U23**  **...........................................**

**U25**  **...........................................**

**U26**  **...........................................**

**U27**  **...........................................**

**U31**  **...........................................**

**U37**  **...........................................**

**U44**  **...........................................**

**U46**  **...........................................**

**U49**  **...........................................**

**U50**  **...........................................**

**U51**  **...........................................**

**U53**  **...........................................**

**U57**  **...........................................**

**U62**  **...........................................**

**U64**  **...........................................**

**U69**  **...........................................**

**U70**  **...........................................**

**U71**  **...........................................**

**U72**  **...........................................**

**U73**  **...........................................**

**U75**  **...........................................**

**U77**  **...........................................**

**U78**  **...........................................**

**U79**  **...........................................**

**U87**  **...........................................**

**U88**  **...........................................**

**U98**  **...........................................**

**U101** **...........................................**

**File S2.** Multiple sequence alignment of the 5’-end of *mba* gene of the reference strain UPA3 and 35 *Ureaplasma* serovar 3 clinical strains.

10 20 30 40 50 60 70 80 90 100 110 120

....|....|....|....|....|....|....|....|....|....|....|....|....|....|....|....|....|....|....|....|....|....|....|....|

**UPA6**  **GTATTTGCAATCTTTATATGTTTTCGTTAAAATTAAAAATTAATTACTATAAAAATTATGTAAGATTAATAAATCTTAGTGTTCATATTTTTTACTAGTATTAAATTAAAAACAATAAAA**

**U1**  **........................................................................................................................**

**U3**  **........................................................................................................................**

**U10**  **........................................................................................................................**

**U19**  **........................................................................................................................**

**U35**  **........................................................................................................................**

**U41**  **................................................G..................................T...........C........................**

**U43**  **................................................G..................................T...........C........................**

**U63**  **........................................................................................................................**

**U80**  **........................................................................................................................**

**U81**  **........................................................................................................................**

**U83**  **........................................................................................................................**

**U85**  **........................................................................................................................**

**U86**  **........................................................................................................................**

**U89**  **........................................................................................................................**

**U91**  **........................................................................................................................**

**U92**  **........................................................................................................................**

**U93**  **........................................................................................................................**

**U99**  **........................................................................................................................**

**U100**  **........................................................................................................................**

130 140 150 160 170 180 190 200 210 220 230 240

....|....|....|....|....|....|....|....|....|....|....|....|....|....|....|....|....|....|....|....|....|....|....|....|

**UPA6**  **TGACATATTTTTTATATTAGGAGAACCATAAATGAAATTATTAAAAAATAAAAAATTCTGAGCTATGACATTAGGAGTTACCTTAGTTGGAGCTGGAATAGTTGCTATAGCGGCTTCATG**

**U1**  **........................................................................................................................**

**U3**  **........................................................................................................................**

**U10**  **........................................................................................................................**

**U19**  **........................................................................................................................**

**U35**  **........................................................................................................................**

**U41**  **.........................T..............................................................................................**

**U43**  **.........................T..............................................................................................**

**U63**  **........................................................................................................................**

**U80**  **........................................................................................................................**

**U81**  **........................................................................................................................**

**U83**  **........................................................................................................................**

**U85**  **........................................................................................................................**

**U86**  **........................................................................................................................**

**U89**  **........................................................................................................................**

**U91**  **........................................................................................................................**

**U92**  **........................................................................................................................**

**U93**  **........................................................................................................................**

**U99**  **........................................................................................................................**

**U100**  **........................................................................................................................**

250 260 270 280 290 300 310 320 330 340 350 360

....|....|....|....|....|....|....|....|....|....|....|....|....|....|....|....|....|....|....|....|....|....|....|....|

**UPA6**  **TTCTAATTCAACTGTTAAATCTAAGTTAAGTAGCCAATTTGTTAAATCAACAGATGATAAAAGTTTTTATGCAGTTTACGAAATTGAAAACTTTAAAGATCTAAGTGATAATGATAAAAA**

**U1**  **........................................................................................................................**

**U3**  **........................................................................................................................**

**U10**  **........................................................................................................................**

**U19**  **........................................................................................................................**

**U35**  **........................................................................................................................**

**U41**  **................................A.G....C................................G...............................................**

**U43**  **................................A.G....C................................G...............................................**

**U63**  **........................................................................................................................**

**U80**  **........................................................................................................................**

**U81**  **........................................................................................................................**

**U83**  **........................................................................................................................**

**U85**  **........................................................................................................................**

**U86**  **........................................................................................................................**

**U89**  **........................................................................................................................**

**U91**  **........................................................................................................................**

**U92**  **........................................................................................................................**

**U93**  **........................................................................................................................**

**U99**  **........................................................................................................................**

**U100**  **........................................................................................................................**

370 380 390 400

....|....|....|....|....|....|....|....|....

**UPA6**  **ATCATTAAATGACATTGAATTTAATGCTGCACTTACATCAGCTG**

**U1**  **............................................**

**U3**  **............................................**

**U10**  **............................................**

**U19**  **............................................**

**U35**  **............................................**

**U41**  **............................................**

**U43**  **............................................**

**U63**  **............................................**

**U80**  **............................................**

**U81**  **............................................**

**U83**  **............................................**

**U85**  **............................................**

**U86**  **............................................**

**U89**  **............................................**

**U91**  **............................................**

**U92**  **............................................**

**U93**  **............................................**

**U99**  **............................................**

**U100**  **............................................**

**File S3.** Multiple sequence alignment of the 5’-end of *mba* gene of the reference strain UPA6 and 19 *Ureaplasma* serovar 6 clinical strains.

10 20 30 40 50 60 70 80 90 100 110 120

....|....|....|....|....|....|....|....|....|....|....|....|....|....|....|....|....|....|....|....|....|....|....|....|

**UUR4**  **GTATTTGCAATCTTTATATGTTTTCGTTAAAATTAAAATTCCTATAAAAACAACATGAGATTAAACAAAATCTTAATGTTGTTGTTATCTATACATTCTAAAGAAAAATATATTTGCAAA**

**UUR10** **........................................................................................................................**

**UUR12** **........................................................................................................................**

**UUR13** **........................................................................................................................**

**U4**  **........................................................................................................................**

**U5**  **........................................................................................................................**

**U11**  **........................................................................................................................**

**U13**  **........................................................................................................................**

**U24**  **........................................................................................................................**

**U29**  **........................................................................................................................**

**U30**  **........................................................................................................................**

**U36**  **........................................................................................................................**

**U42**  **........................................................................................................................**

**U45**  **........................................................................................................................**

**U47**  **........................................................................................................................**

**U48**  **........................................................................................................................**

**U54**  **........................................................................................................................**

**U56**  **........................................................................................................................**

**U66**  **........................................................................................................................**

**U67**  **........................................................................................................................**

**U74**  **........................................................................................................................**

**U90**  **........................................................................................................................**

**U95**  **........................................................................................................................**

130 140 150 160 170 180 190 200 210 220 230 240

....|....|....|....|....|....|....|....|....|....|....|....|....|....|....|....|....|....|....|....|....|....|....|....|

**UUR4**  **ACTATAAATAGACACAAAAAACAATAGAATAATAAAACTAAATTTCGTATTTAGTTTATTAGGAGATCGTTATAAATGAAATTATTAAAAAATAAGAAATTTTGAGCAATTACACTAGGG**

**UUR10** **........................................................................................................................**

**UUR12** **........................................................................................................................**

**UUR13** **........................................................................................................................**

**U4**  **........................................................................................................................**

**U5**  **........................................................................................................................**

**U11**  **...............................................................................................A........................**

**U13**  **........................................................................................................................**

**U24**  **........................................................................................................................**

**U29**  **........................................................................................................................**

**U30**  **........................................................................................................................**

**U36**  **........................................................................................................................**

**U42**  **........................................................................................................................**

**U45**  **........................................................................................................................**

**U47**  **........................................................................................................................**

**U48**  **........................................................................................................................**

**U54**  **........................................................................................................................**

**U56**  **........................................................................................................................**

**U66**  **........................................................................................................................**

**U67**  **........................................................................................................................**

**U74**  **........................................................................................................................**

**U90**  **........................................................................................................................**

**U95**  **........................................................................................................................**

250 260 270 280 290 300 310 320 330 340 350 360

....|....|....|....|....|....|....|....|....|....|....|....|....|....|....|....|....|....|....|....|....|....|....|....|

**UUR4**  **GTAACTTTAGTGGGAGCAGGGGTAGTTGCTGTGGCAGCTTCATGTTCTAGCTCAAATGTTAAATCTAAATTAAGTAGTCAACTTGTTAAATCAAAAGACGAAAAGAGCTTTTACGCTGTT**

**UUR10** **........................................................................................................................**

**UUR12** **........................................................................................................................**

**UUR13** **........................................................................................................................**

**U4**  **........................................................................................................................**

**U5**  **........................................................................................................................**

**U11**  **........................................................................................................................**

**U13**  **........................................................................................................................**

**U24**  **........................................................................................................................**

**U29**  **........................................................................................................................**

**U30**  **........................................................................................................................**

**U36**  **........................................................................................................................**

**U42**  **........................................................................................................................**

**U45**  **........................................................................................................................**

**U47**  **........................................................................................................................**

**U48**  **........................................................................................................................**

**U54**  **........................................................................................................................**

**U56**  **........................................................................................................................**

**U66**  **........................................................................................................................**

**U67**  **........................................................................................................................**

**U74**  **........................................................................................................................**

**U90**  **........................................................................................................................**

**U95**  **........................................................................................................................**

370 380 390 400 410 420 430 440

....|....|....|....|....|....|....|....|....|....|....|....|....|....|....|....|....|...

**UUR4**  **TACGACATTGAAAATTTCGATGATTTAAATGAAAATGATAAAAAAGCATTAAACGAAGCTGAATTTAATGCTGCACTTACATCAGCTG**

**UUR10** **........................................................................................**

**UUR12** **........................................................................................**

**UUR13** **........................................................................................**

**U4**  **........................................................................................**

**U5**  **........................................................................................**

**U11**  **........................................................................................**

**U13**  **........................................................................................**

**U24**  **........................................................................................**

**U29**  **........................................................................................**

**U30**  **........................................................................................**

**U36**  **........................................................................................**

**U42**  **........................................................................................**

**U45**  **........................................................................................**

**U47**  **........................................................................................**

**U48**  **........................................................................................**

**U54**  **........................................................................................**

**U56**  **........................................................................................**

**U66**  **........................................................................................**

**U67**  **........................................................................................**

**U74**  **........................................................................................**

**U90**  **........................................................................................**

**U95**  **........................................................................................**

**File S4.** Multiple sequence alignment of the 5’-end of *mba* gene of the reference strains UUR4, UUR10, UUR12, UUR13 and 19 *Ureaplasma* serovars 4, 10, 12, 13 clinical strains.

10 20 30 40 50 60 70 80 90 100 110 120

....|....|....|....|....|....|....|....|....|....|....|....|....|....|....|....|....|....|....|....|....|....|....|....|

**UUR2** **GTATTTGCAATCTTTATATGTTTTCGTTAAAATTAAAATTCCTATTAAAAACAACATGAGATTAAACAAAATCTTAATGTTGTTATTATCTATACATTCTAAAGAAAAATATATTTGCAA**

**UUR5** **........................................................................................................................**

**UUR8** **........................................................................................................................**

**UUR9** **........................................................................................................................**

**U21**  **........................................................................................................................**

**U22**  **........................................................................................................................**

**U33**  **........................................................................................................................**

**U34**  **........................................................................................................................**

**U40**  **......................................................T.................................................................**

**U52**  **........................................................................................................................**

**U55**  **........................................................................................................................**

**U61**  **........................................................................................................................**

**U84**  **........................................................................................................................**

130 140 150 160 170 180 190 200 210 220 230 240

....|....|....|....|....|....|....|....|....|....|....|....|....|....|....|....|....|....|....|....|....|....|....|....|

**UUR2** **AACTATAAATAGACACAAAAAACAATAGAATAATAAAACTAAATTTCATATTTAGTTTATTAGGAGATCGTTATAAATGAAATTATTAAAAAATAAGAAATTTTGAGCAATTACACTAGG**

**UUR5** **........................................................................................................................**

**UUR8** **........................................................................................................................**

**UUR9** **........................................................................................................................**

**U21**  **........................................................................................................................**

**U22**  **........................................................................................................................**

**U33**  **........................................................................................................................**

**U34**  **........................................................................................................................**

**U40**  **...............................................G........................................................................**

**U52**  **........................................................................................................................**

**U55**  **........................................................................................................................**

**U61**  **........................................................................................................................**

**U84**  **........................................................................................................................**

250 260 270 280 290 300 310 320 330 340 350 360

....|....|....|....|....|....|....|....|....|....|....|....|....|....|....|....|....|....|....|....|....|....|....|....|

**UUR2** **GGTAACTTTAGTGGGAGCAGGGGTAGTTGCTGTGGCAGCTTCATGTTCTAGCTCAAATGTTAAATCTAAATTAAGTAGTCAACTTGTTAAATCAAAAGACGAAAAGAGCTTTTACGCTGT**

**UUR5** **........................................................................................................................**

**UUR8** **........................................................................................................................**

**UUR9** **........................................................................................................................**

**U21**  **........................................................................................................................**

**U22**  **........................................................................................................................**

**U33**  **........................................................................................................................**

**U34**  **........................................................................................................................**

**U40**  **........................................................................................................................**

**U52**  **........................................................................................................................**

**U55**  **........................................................................................................................**

**U61**  **........................................................................................................................**

**U84**  **........................................................................................................................**

370 380 390 400 410 420 430 440

....|....|....|....|....|....|....|....|....|....|....|....|....|....|....|....|....|....

**UUR2** **TTACGACATTGAAAATTTCGATGATTTAACTGAAAATGATAAAAAAGCATTAAACGAAGCTGAATTTAATGCTGCACTTACATCAGCTG**

**UUR5** **.........................................................................................**

**UUR8** **.........................................................................................**

**UUR9** **.........................................................................................**

**U21**  **.........................................................................................**

**U22**  **.........................................................................................**

**U33**  **.........................................................................................**

**U34**  **.........................................................................................**

**U40**  **.........................................................................................**

**U52**  **.........................................................................................**

**U55**  **.........................................................................................**

**U61**  **.........................................................................................**

**U84**  **.........................................................................................**

**File S5.** Multiple sequence alignment of the 5’-end of *mba* gene of the reference strains UUR2, UUR5, UUR8, UUR9 and 19 *Ureaplasma* serovars 2, 5, 8, 9 clinical strains.

50 60 70 80 90

|....|....|....|....|....|....|....|....|....|

**ParC_consensus** **AMSELGIFHDKPYKKSARTVGEVIGKYHPHGDSSIYEAMVRMSQD**

**U2**  **.............................................**

**U4**  **.............................................**

**U6**  **.............................................**

**U16**  **.............................................**

**U1**  **................................L............**

**U10**  **................................L............**

**U11**  **................................L............**

**U13**  **................................L............**

**U14**  **................................L............**

**U15**  **................................L............**

**U21**  **................................L............**

**U22**  **................................L............**

**U24**  **................................L............**

**U30**  **................................L............**

**U33**  **................................L............**

**U34**  **................................L............**

**U36**  **................................L............**

**U40**  **................................L............**

**U47**  **................................L............**

**U52**  **................................L............**

**U54**  **................................L............**

**U55**  **................................L............**

**U56**  **................................L............**

**U66**  **................................L............**

**U67**  **................................L............**

**U84**  **................................L............**

**File S6.** Partial sequence alignment of ParC protein of 26 *Ureaplasma* isolates resistant to fluoroquinolones (ParC_consensus correspond to ParC protein of UPA3 and UUR8 reference strains).

430 440 450 460 470 480

....|....|....|....|....|....|....|....|....|....|....|....|

**ParE_consensus** **LFLVEGDSAGGSAKLGRNKKYQAILPLRGKVLNVLKARLVDVLKNEEIASIFTCLGTGIG**

**U21**  **.........................S..................................**

**U55**  **.........................S..................................**

**U84**  **.........................S..................................**

**U1**  **............................................................**

**U2**  **............................................................**

**U4**  **............................................................**

**U6**  **............................................................**

**U10**  **............................................................**

**U11**  **............................................................**

**U13**  **............................................................**

**U14**  **............................................................**

**U15**  **............................................................**

**U16**  **............................................................**

**U22**  **............................................................**

**U24**  **............................................................**

**U30**  **............................................................**

**U33**  **............................................................**

**U34**  **............................................................**

**U36**  **............................................................**

**U40**  **............................................................**

**U47**  **............................................................**

**U52**  **............................................................**

**U54**  **............................................................**

**U56**  **............................................................**

**U66**  **............................................................**

**U67**  **............................................................**

**File S7.** Partial sequence alignment of ParE protein of 26 *Ureaplasma* isolates resistant to fluoroquinolones (ParE_consensus correspond to ParE protein of UPA3 and UUR8 reference strains).

110 120 130 140 150 160

....|....|....|....|....|....|....|....|....|....|....|....|....

**L22_consensus** **TTHLEIVLSDDVNEREKELAAIKAKKSKKPLAVEPIAKVETKKVAKPSKVEIKPVEKDENVDPE**

**U1**  **....................S...................I.......................**

**U2**  **....................S...................I.......................**

**U4**  **....................S...................I.......................**

**U6**  **....................S...................I.......................**

**U10**  **....................S...................I.......................**

**U11**  **....................S...................I.......................**

**U13**  **....................S...................I.......................**

**U14**  **....................S...................I.......................**

**U15**  **....................S...................I.......................**

**U16**  **....................S...................I.......................**

**U21**  **....................S...................I.......................**

**U22**  **....................S...................I.......................**

**U24**  **....................S...................I.......................**

**U30**  **....................S...................I.......................**

**U33**  **....................S...................I.......................**

**U34**  **....................S...................I.......................**

**U36**  **....................S...................I.......................**

**U40**  **....................S...................I.......................**

**U47**  **....................S...................I.......................**

**U52**  **....................S...................I.......................**

**U54**  **....................S...................I.......................**

**U55**  **....................S...................I.......................**

**U56**  **....................S...................I.......................**

**U66**  **....................S...................I.......................**

**U67**  **....................S...................I.......................**

**U84**  **....................S...................I.......................**

**File S8.** Partial sequence alignment of L22 protein of 26 *Ureaplasma* isolates resistant to macrolides (L22_consensus correspond to L22 protein of UPA3 and UUR8 reference strains).
